# TopoRoot: a method for computing hierarchy and fine-grained traits of maize roots from 3D imaging

**DOI:** 10.1186/s13007-021-00829-z

**Published:** 2021-12-13

**Authors:** Dan Zeng, Mao Li, Ni Jiang, Yiwen Ju, Hannah Schreiber, Erin Chambers, David Letscher, Tao Ju, Christopher N. Topp

**Affiliations:** 1 Department of Computer Science and Engineering, Washington University in St. Louis, Saint Louis, MO 63130 USA; 2 Donald Danforth Plant Science Center, Saint Louis, MO 63132 USA; 3 Department of Computer Science, Saint Louis University, Saint Louis, MO 63103 USA

**Keywords:** Root system architecture, Phenotyping, 3D Imaging, Topology, Computer Graphics

## Abstract

**Background:**

3D imaging, such as X-ray CT and MRI, has been widely deployed to study plant root structures. Many computational tools exist to extract coarse-grained features from 3D root images, such as total volume, root number and total root length. However, methods that can accurately and efficiently compute fine-grained root traits, such as root number and geometry at each hierarchy level, are still lacking. These traits would allow biologists to gain deeper insights into the root system architecture.

**Results:**

We present TopoRoot, a high-throughput computational method that computes fine-grained architectural traits from 3D images of maize root crowns or root systems. These traits include the number, length, thickness, angle, tortuosity, and number of children for the roots at each level of the hierarchy. TopoRoot combines state-of-the-art algorithms in computer graphics, such as topological simplification and geometric skeletonization, with customized heuristics for robustly obtaining the branching structure and hierarchical information. TopoRoot is validated on both CT scans of excavated field-grown root crowns and simulated images of root systems, and in both cases, it was shown to improve the accuracy of traits over existing methods. TopoRoot runs within a few minutes on a desktop workstation for images at the resolution range of 400^3, with minimal need for human intervention in the form of setting three intensity thresholds per image.

**Conclusions:**

TopoRoot improves the state-of-the-art methods in obtaining more accurate and comprehensive fine-grained traits of maize roots from 3D imaging. The automation and efficiency make TopoRoot suitable for batch processing on large numbers of root images. Our method is thus useful for phenomic studies aimed at finding the genetic basis behind root system architecture and the subsequent development of more productive crops.

**Supplementary Information:**

The online version contains supplementary material available at 10.1186/s13007-021-00829-z.

## Introduction

Roots are the primary means by which the plant absorbs water and nutrients, and they provide anchorage to the plant. These functions are largely determined by the root system architecture (RSA) [1–3], which describes both the geometry of individual roots and their hierarchical relationships. Quantifying RSA enables efforts to discover the genetic control of root traits, which can lead to improved crop productivity while minimizing adverse environmental effects [2, 4]. However, RSA is difficult to study owing to roots’ poor accessibility as the “hidden half” of the plant. Traditionally, roots are excavated from the soil, washed, and then measured by hand using devices such as rulers, calipers, and protractors. This process is not only labor-intensive but also prone to human errors. More importantly, many aspects of RSA, particularly those pertaining to lateral roots of higher order, are almost impossible to measure by hand.

Advances in 3D imaging, including X-ray CT, MRI, and optical imaging [5–7], have allowed root shapes to be captured digitally either after excavation or in situ. The availability of such imaging data has paved the way for recent efforts towards computational quantification of root system architecture [8–10]. However, most image-based root phenotyping methods only compute overall traits such as the volume, depth, convex hull volume, total root length, and root number [11–14]. Though useful, these traits which are aggregated over the whole root system do not capture the branching structure or the hierarchical organization of individual roots, which provide a much more comprehensive description of RSA. Recently, a system for multi-view scanning and subsequent computational analysis, known as DIRT/3D [15], was proposed to measure detailed traits of maize root crowns. The system can report 18 traits that concern the geometry of the stem (e.g., diameter and depth), the upper whorls (e.g., inter-whorl distance) and their individual nodal roots (e.g., length, angle and diameter). An inherent challenge for multi-view reconstruction, due to occlusion, is resolving densely packed roots. As a result, DIRT/3D does not provide a full root hierarchy beyond the nodal roots.

To our knowledge, DynamicRoots [16] is the only published and validated root phenotyping method that produces a full branching hierarchy and root traits associated with each hierarchy level in 3-dimensions. DynamicRoots is designed for a time-series of root systems grown in transparent gel [17]. These seedling-stage root systems tend to have a relatively simple geometry and structure, which makes it possible to obtain high quality 3D voxelized reconstructions using multi-view imaging [18]. DynamicRoots first employs graph analysis on the voxelized root system at each time-point to identify the root branches. Hierarchical relations among the branches are first determined by the length of the branches and then refined by the time function obtained by aligning root architectures across time. While, in theory, DynamicRoots can process any segmented 3D root image, its accuracy can be significantly affected by the number of topological errors in the segmentation, such as disconnected root components and root branches forming loops due to touchings. Although such errors are scarce in the multi-review reconstruction of simple seedling-stage roots, they can be abundant in 3D images of more complex root systems. In addition, DynamicRoots requires a time series to obtain the correct root hierarchy.

In this work, we present TopoRoot, a method for obtaining the complete root hierarchy and associated fine-grained traits of a mature maize root system (or crown) from a single 3D image. Compared with DynamicRoots, TopoRoot is designed to deal with topological errors, which are common in images of complex root systems, and to infer the hierarchy without the need for a time series. TopoRoot builds on several state-of-the-art algorithms from computer graphics, including topological simplification and 3D skeletonization, and introduces customized heuristics tailored to the maize root structure.

TopoRoot is validated on both real and simulated data. On a set of 45 X-ray CT scans of excavated maize root crowns, TopoRoot shows dramatic improvements in accuracy over DynamicRoots in counting the number of nodal roots. On another set of 495 synthetically generated images of maize root systems simulated by OpenSimRoot [19] with varying age, complexity, and noise level, TopoRoot exhibits improved accuracy in a variety of coarse-grained and fine-grained traits over DynamicRoots and GiaRoots [14].

TopoRoot is completely automated and requires setting only three thresholds for each image. On a standard desktop computer, TopoRoot runs within a few minutes for images at the resolution range of 400^3. This makes TopoRoot suited for batch processing a large set of images in a high-throughput analysis pipeline. The software package is freely distributed on GitHub with our X-ray CT dataset.

## Methods

### Overview

**Fig. 1:**
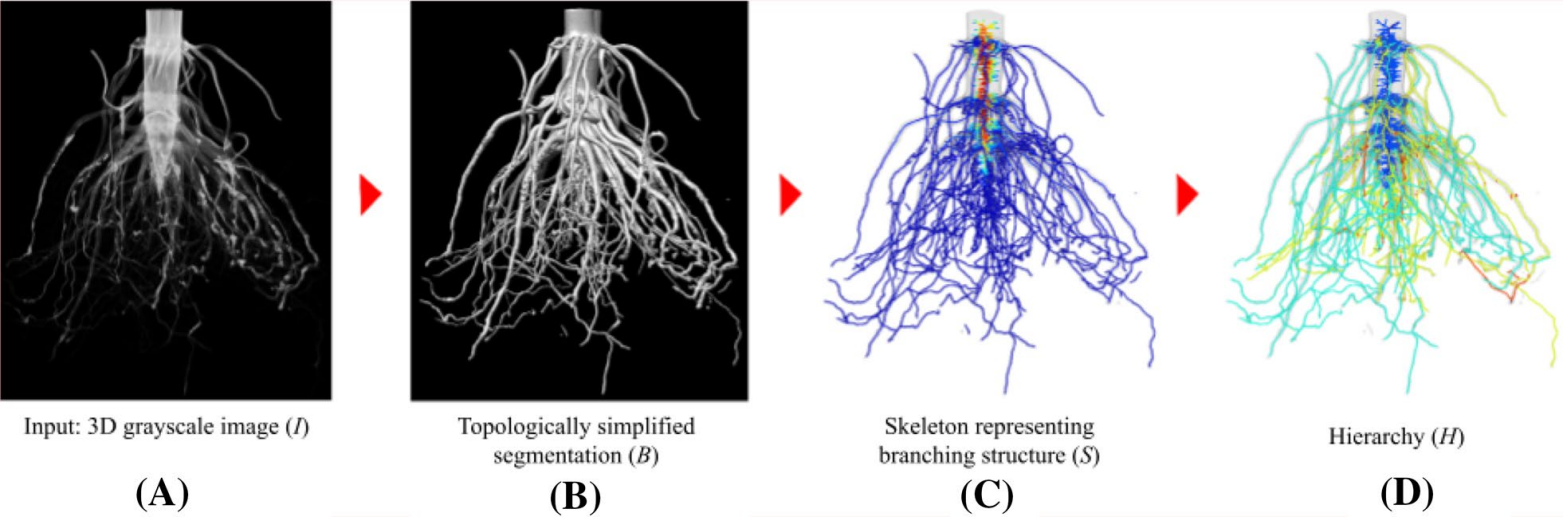
The pipeline of TopoRoot for computing fine-grained traits from a 3D image. Beginning from a 3D grayscale image $I$ represented as a stack of image slices (**A**), TopoRoot first computes a topologically simple segmentation $B$ (**B**), then it creates a geometric skeleton $S$ capturing the branching structure (**C**), from which a hierarchy $H$ is obtained (**D**) and the traits are subsequently computed. This example and the ones in Figs. 2, 3, 4 are taken from our CT scans of root crowns; see details in "Results" section

The input to the TopoRoot pipeline is a 3D grayscale image, $I$, of a maize root crown or root system, represented as a stack of 2D image slices. The pipeline assumes that $I$ has sufficient contrast between the roots and their surroundings (e.g., soil), so that intensity thresholding can be used to segment the roots. If this assumption does not hold, such as when large chunks of soil are attached to the roots or in the case of in situ imaging in soil, TopoRoot may still be applied if the roots can be segmented by a third-party segmentation algorithm (see "Discussions" section). TopoRoot produces a root hierarchy and fine-grained root traits in four steps (see Fig. 1).

**Fig. 2:**
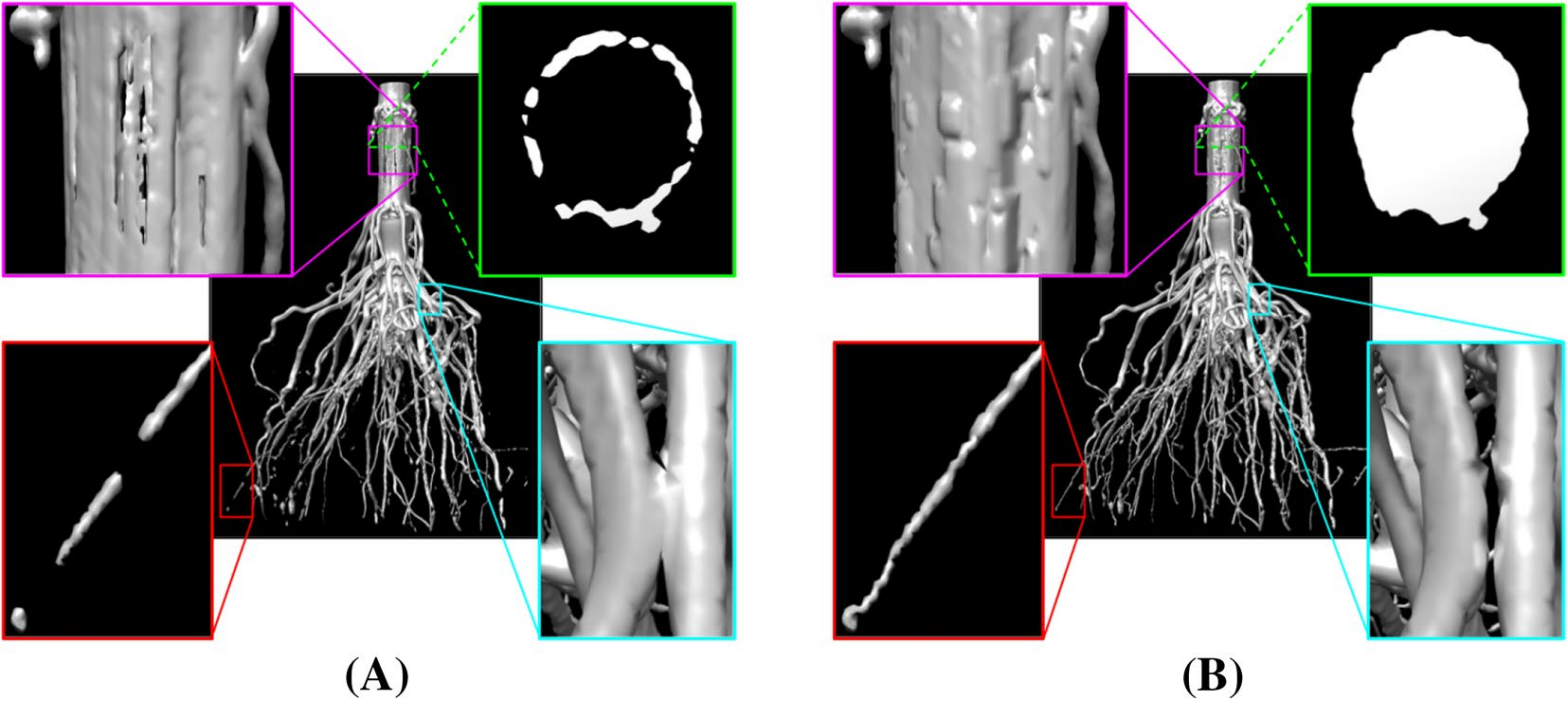
Segmenting a root image. **A** Thresholding the image $I$ yields numerous topological errors such as disconnections (red box) and handles (cyan and purple boxes), and the stem has a hollow interior (green box). **B** Applying our filling heuristic followed by the algorithm of [20] yields a segmentation $B$ with these topological errors removed and the stem filled

**Fig. 3:**
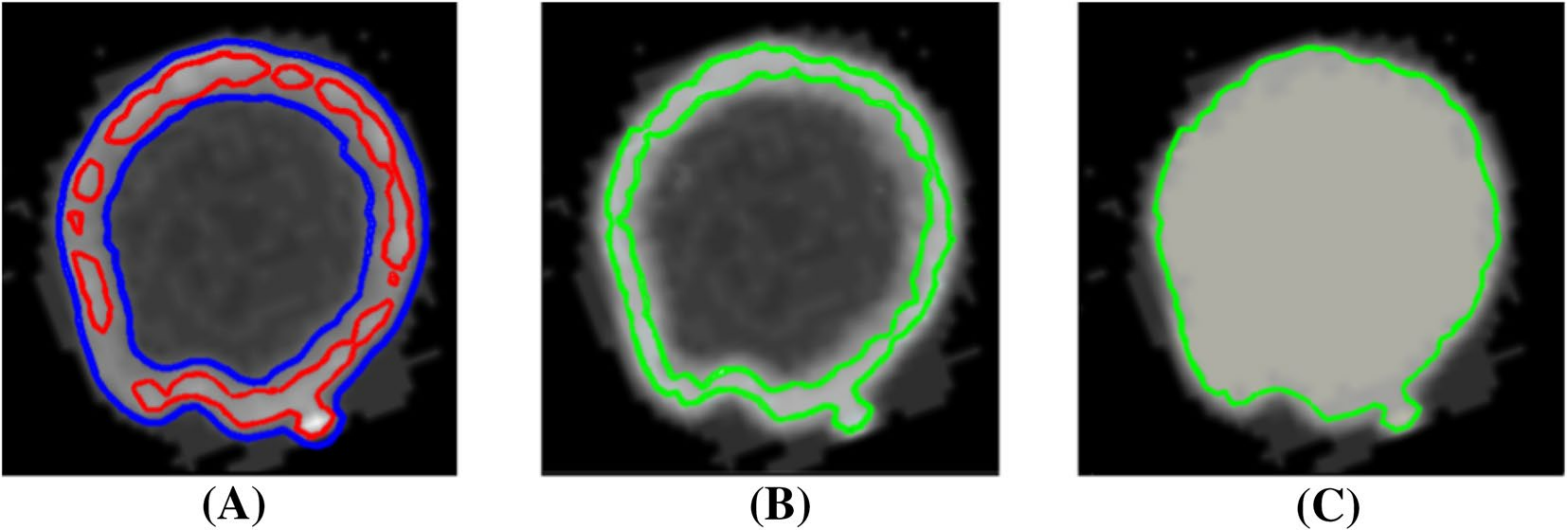
Filling the hollow space inside the stem and thick roots. **A** A slice of $I$ showing the cross-section of the stem with the outline of segmentations $B_{mid} ,B_{low}$ (red and blue) at thresholds $t_{mid} ,t_{low}$. The hollow space within the stem is connected to the outside in $B_{mid}$ but is closed off by the stem’s shell in $B_{low}$. **B** Eroding $B_{low}$ onto $B_{mid}$, while preserving its topology, results in the shape $B_{mid} ^{\prime}$(green), which adds a minimal set of voxels to $B_{mid}$ to “close off” the hollow space. **C** The hollow space is filled by raising the intensity value of the voxels in the void of $B_{mid} ^{\prime}$ and the voxels in $B_{mid} ^{\prime}\backslash B_{mid}$ adjacent to the void to $B_{mid}$. This creates a new image $I^{\prime}$

1. **Segmentation**: This step produces a binary segmentation, $B$, from image $I$ that captures the root shape with few topological errors. It does so by combining a recently developed topological simplification algorithm [20] with a new algorithm that ensures the solidity of the root stem
2. **Skeletonization**: This step computes a skeleton, $S$, from the binary segmentation $B$ such that $S$ has the correct topology of a tree (that is, being connected and free of cycles). This is done by employing an existing skeletonization algorithm [21, 22] followed by a novel heuristic to remove cycles.
3. **Inferring hierarchy**: A hierarchy, $H$, is obtained from the skeleton $S$ so that each edge of $S$ is given an integer indicating its level in the hierarchy (e.g., 0 means the stem, 1 means the nodal roots, 2 means the lateral roots of first order, etc.). The hierarchy algorithm minimizes the depth of the hierarchy while favoring longer branches at lower levels.
4. **Computing traits**: Finally, a suite of root traits, such as the count, lengths, angles, thickness, and tortuosity, are computed from the skeleton $S$ at each level of the hierarchy $H$.

These steps are detailed in the next few sections.

### Segmentation

Each maize root system has a simple topology: it is connected, free of handles (“loops”) or voids (“air bubbles”). However, due to limits in imaging resolution, contrast, and/or noise level, simple thresholding of the input grayscale image often yields a segmentation with numerous disconnected components, handles and voids (see Fig. 2A). These erroneous topological features pose significant challenges for the subsequent hierarchy inference. Another issue with simple thresholding is that due to the relatively low intensity in the interior of thick roots (e.g., the stem), only the outer shell of these roots is included in the segmentation (see Fig. 2A). These hollow shells would lead to complex skeletons that do not accurately capture the tubular shape of the roots. Given an input image $I$, the first step extracts a segmentation $B$ that fills the hollow root interior and has minimal topological errors.

We start by filling the hollow interior of roots using an erosion approach. The observation is that these hollow spaces become voids (i.e., closed off by the root’s shell) when the image $I$ is thresholded by a sufficiently low value. Our heuristic identifies these voids at a low threshold and fills them in after “growing” them back to the normal threshold. The heuristic takes in two user-specified thresholds, $t_{mid}$ and $t_{low}$, such that $t_{mid}$ best captures of the shape of the root while $t_{low} < t_{mid}$ closes off most of the hollow spaces within roots. We denote the segmented, voxelized shapes at these two thresholds as $B_{mid}$ and $B_{low}$ respectively (see Fig. 3A). To grow the voids in $B_{low}$ back to the hollow spaces in $B_{mid}$, we maximally erode the voxels in $B_{low}$ while maintaining its topology and preventing voxels in $B_{mid}$ from being eroded. This results in another shape, denoted as $B_{mid} ^{\prime}$, which is a minimal superset of $B_{mid}$ with its hollow spaces closed off (see Fig. 3B). We then take all voxels in the voids of $B_{mid} ^{\prime}$, together with those voxels in $B_{mid} ^{\prime}\backslash B_{mid}$ adjacent to the voids, and “fill” them by setting their intensity values to be $t_{mid}$ (see Fig. 3C). We denote the resulting image as $I^{\prime}$.

**Fig. 4:**
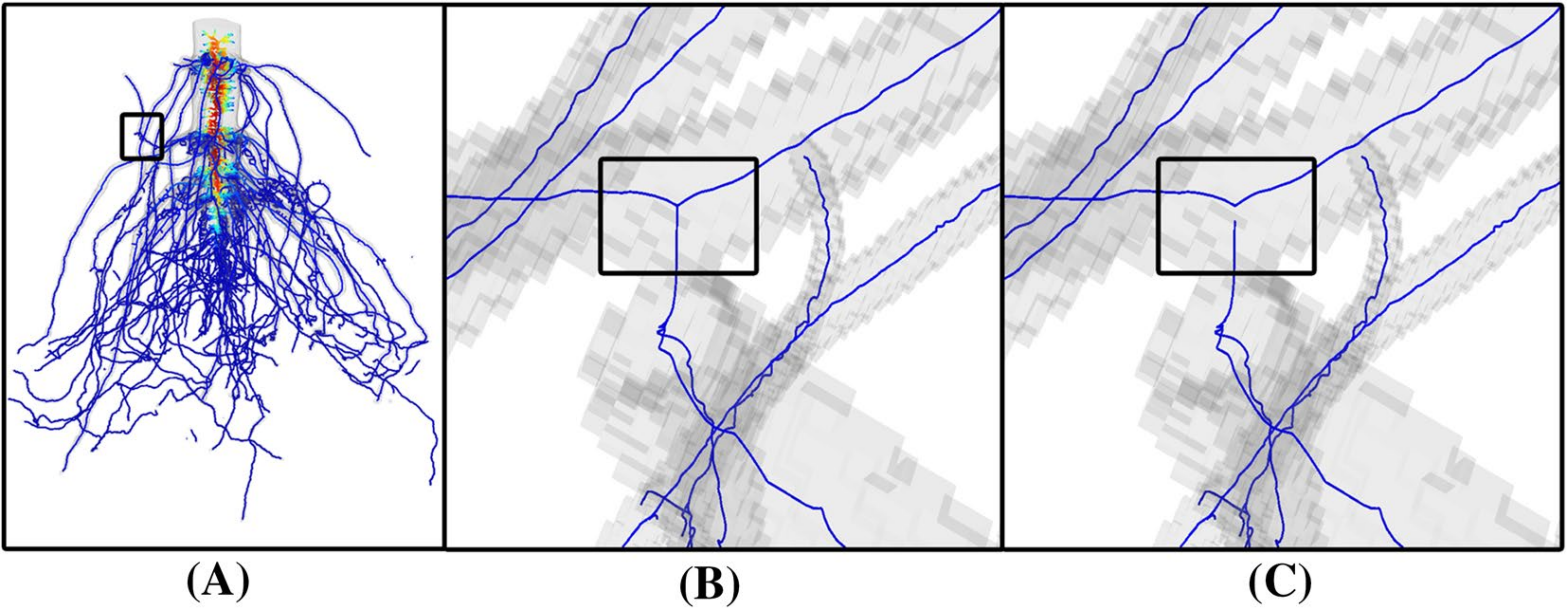
Computing a cycle-free skeleton. **A** An initial curve skeleton ${S}_{0}$ computed by the methods of [21, 22], where color indicates the thickness of the roots (redder curves lie in thicker roots). **B** A skeleton junction (in the boxed region in (A)) caused by two roots touching in the segmentation, which leads to a cycle in the skeleton. **C** The same region on the final skeleton $S$ where the cycles have been removed using a graph-based algorithm

Next, we compute a segmentation of the hollow-space-filled image $I^{\prime}$ using the algorithm of [20]. This algorithm uses global optimization to extract a segmentation bounded between two intensity thresholds that has the least number of topological features. It takes three thresholds with increasing values, $t_{low} < t_{mid} < t_{high}$, where $t_{low} ,t_{mid}$ are the same as in the filling algorithm above. Let the segmented shapes of $I^{\prime}$ at these thresholds be $B_{low} ,B_{mid} ,B_{high}$. The algorithm of [20] computes a shape $B$ such that $B_{high} \subseteq B \subseteq B_{low}$ and the following vector energy is minimal in lexicographical order,

1 $$\left\{ {\beta_{0} \left( B \right) + \beta_{1} \left( B \right) + \beta_{2} \left( B \right), diff\left( {B,B_{mid} } \right)} \right\}$$

Here, $\beta_{0} ,\beta_{1} ,\beta_{2}$ counts the number of connected components, handles and voids of $B$, and $diff$ is a difference measure between two voxel sets that considers both the number and intensity of voxels that are in one of the sets but not the other. Intuitively, $B$ makes the least change to the shape $B_{mid}$ (in terms of $diff$) to remove as many topological features on $B_{mid}$ as possible while sandwiched between $B_{high}$ and $B_{low}$.

An example result of this step (filling hollow spaces and then applying the algorithm of [20]) is shown in Fig. 2B. The three thresholds $t_{low} , t_{mid} , t_{high}$ control the trade-off between topological simplicity and geometric fidelity of the segmentation $B$. A larger gap between $t_{mid}$ and $t_{low} , t_{high}$ gives the algorithm [20] more room to remove topological features, and hence the result has fewer topological errors. But this comes at the cost of possibly large and undesirable geometric changes; for example, a root may be broken in the middle to remove a topological handle. We found that the best results are obtained by setting $t_{low}$ to be the highest value such that thin roots remain connected in $B_{low}$ and setting $t_{high}$ to be the lowest value before roots start to merge in $B_{high}$. The resulting segmentation $B$ will not be completely free of topological errors but fixing these remaining errors in a geometrically correct way requires a more global context of the root shape. This will be addressed in the next step and with the help of a geometric skeleton.

### Skeletonization

The tubular shape of roots makes them representable by curve skeletons. The graph structure of a skeleton is the key that enables subsequent analysis of branching hierarchy and traits. Given the segmentation $B$ produced by the previous step, this step produces a geometric skeleton $S$ capturing the shape and branching structure of the roots. We will utilize the structural information provided by the skeleton to resolve the topological errors that remain from the previous step, so that $S$ is connected and free of cycles.

We first compute an initial curve skeleton $S_{0}$ from the voxel shape $B$ using the algorithms described in [21, 22, 34]. These methods have been recently used in skeleton-based phenotyping of sorghum panicles [23]. Specifically, the Voxel Core method of [22] extracts an approximate medial axis of $B$, which is a triangulated 2-dimensional non-manifold that lies at the center of $B$. Taking the medial axis as the input, the Erosion Thickness method of [21] reduces it to a polygonal 1-dimensional skeleton $S_{0}$. Compared with other means for computing skeletons, this approach has several features important for root analysis. Both methods [21, 22] are robust to irregular shape boundaries, and hence spurious skeleton branches are minimized. Both methods also preserve the topology of $B$ exactly, and hence $S_{0}$ carries the same set of topological features as $B$ without adding new features. Both methods are highly optimized and capable of processing large 3D images. Unlike methods that produce skeletons made up of voxels, $S_{0}$ is made up of vertices and edges, and hence it can be conveniently processed by graph algorithms. Finally, the method of [21] also outputs a “thickness” measure for each edge of $S_{0}$, which will be utilized later. An example of this initial skeleton is shown in Fig. 4A (also in Fig. 1C).

**Fig. 5:**
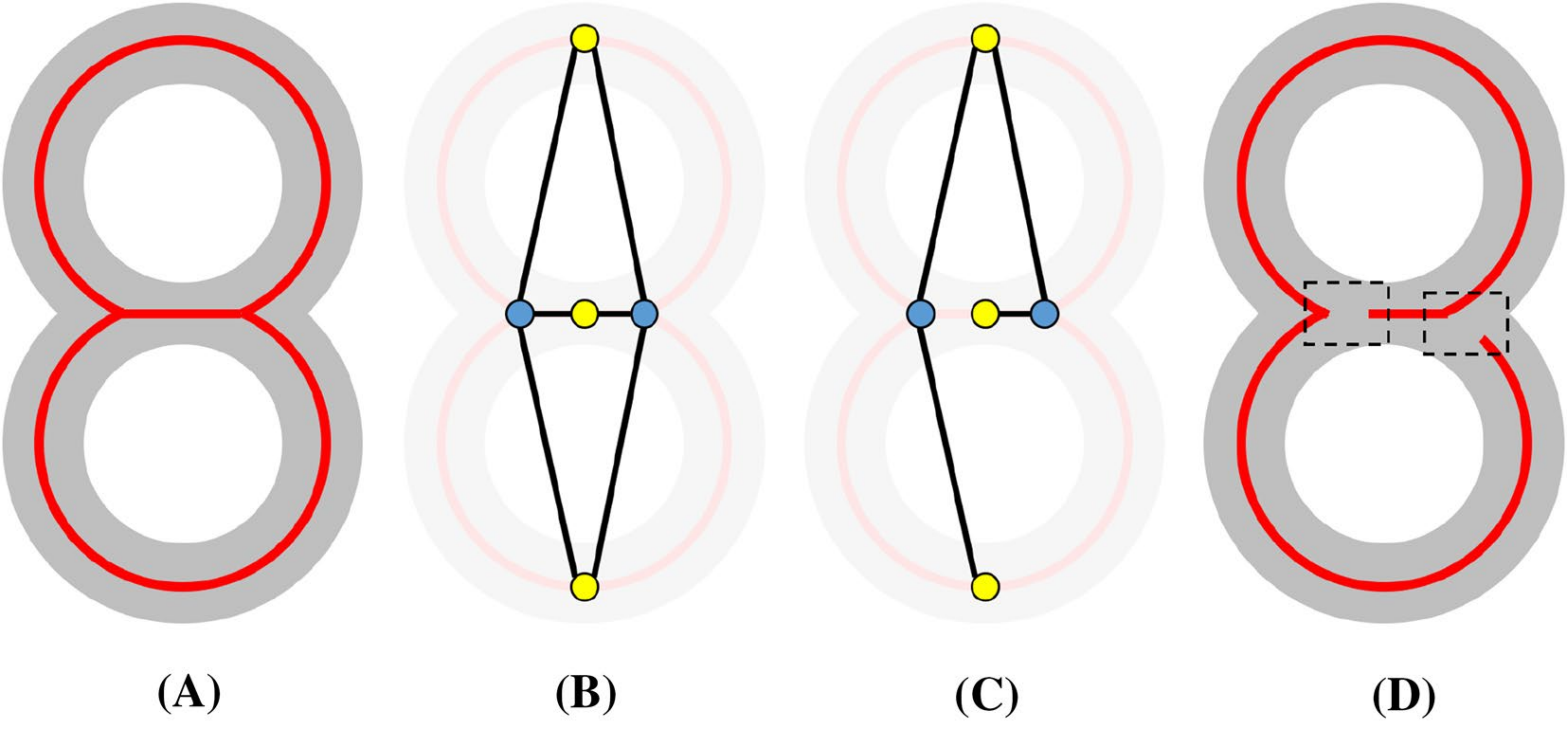
Illustration of the graph-based algorithm for cycle removal. **A** A synthetic segmentation (gray) and its skeleton with two cycles (red). **B** A graph $G$ where each node represents either a skeleton junction (blue) or a skeleton branch (yellow) and each arc (black) connects two nodes representing a junction and an adjacent branch. **C** A spanning tree of $G$ excludes two arcs. **D** The resulting skeleton after detaching two junction-branch pairs (dashed boxes) corresponding to the excluded arcs in **C**. Note that the spanning tree is not unique, and the one shown in **C** is just an example and not necessarily the optimal one

The remaining topological features on the segmentation $B$ manifest as disconnected components and cycles on the initial skeleton $S_{0}$. Figure 4B shows an example of a cycle caused by two touching roots. To remove these features, we take the largest component of $S_{0}$, denoted by $S_{1}$, and remove cycles in $S_{1}$ using a graph-based approach. We first construct a graph $G = \left\{ {N,A} \right\}$ with nodes $N$ and arcs $A$ as follows: each node represents either a *junction* (a vertex incident to three or more skeleton edges) or a *branch* (a sequence of skeleton edges between two junctions or between a junction and an end vertex) on $S_{1}$, and each arc connects two nodes representing a junction and a branch at that junction (see Fig. 5A, B). Next, we extract a *spanning tree* of $G$, which is a subset of arcs $A^{\prime} \subseteq A$ that connect all nodes in $N$ and have no cycles (see Fig. 5C). The final skeleton, $S$, is then obtained from $A^{\prime}$ as follows: for each arc $a \in A$ that does not exist in $A^{\prime}$, the pair of skeleton branch and junction presented by $a$ is “detached” from each other (see Fig. 5D). Note that cycle-removal using this approach prevents a skeleton branch from being broken in the middle.

To find a spanning tree that best captures the structure of the root, we associate each arc $a \in A$ with a positive weight $w\left( a \right)$ and compute the spanning tree $A^{\prime}$ with the minimal total arc weights. $A^{\prime}$ is known as the *minimal spanning tree (MST)* of $G$, and it can be computed efficiently using standard algorithms such as Prim’s or Kruskal’s. The weight $w\left( a \right)$ measures the likelihood that the pair of skeleton junction and branch represented by $a$ should be detached. It is defined as:

2 $$w\left( a \right) = w_{angle} \left( a \right) + \lambda w_{dis} \left( a \right)$$

The first term $w_{angle} \left( a \right)$ measures the continuity of skeleton orientation at the junction. Let $j, b$ be the skeleton junction and branch represented by the arc $a$, $\Omega$ be the set of all branches at $j$, and $\vec{b}$ be the unit vector representing the tangent direction of the branch $b$ oriented towards $j$. The angle term is defined as:

3 $$w_{angle} \left( a \right) = \mathop {min}\limits_{b^{\prime} \in \Omega } \left( {1 + \vec{b} \cdot \overrightarrow {b^{\prime}} } \right)$$

This term reaches the minimum of 0 if there is some other branch $b^{\prime}$ at junction $j$ that has the same tangent direction as $b$. The second term $w_{dis} \left( a \right)$ measures the distance from the junction $j$ represented by the arc $a$ to the root stem. This term exists to discourage detaching branches representing nodal roots from the stem, which would have a great impact on the branching hierarchy. We use the algorithm reported in [23] to identify the stem as the longest non-branching sequence of skeleton edges on the skeleton $S_{1}$ whose thickness measure is above a given threshold $\in$, which was set in our experiments to be 0.15 multiplied by the maximum thickness among any vertex in $S_{1}$. We then compute $w_{dis} \left( a \right)$ as the shortest distance on the skeleton between junction $j$ and any vertex on the stem. Finally, $\lambda$ is a balancing parameter between the angle and distance terms. We found that a small value such as 0.05 best satisfies the need for avoiding detachment of nodal roots without overpowering the angle term $w_{angle} \left( a \right)$. Figure 4C shows the skeleton obtained from the MST of the weighted graph. Observe that the touching between two roots in the highlighted view are correctly detached.

### Inferring hierarchy

Given the cycle-free skeleton $S$ obtained from the previous step, we next label each vertex of the skeleton as either part of the stem, a nodal root, or a lateral root of a specific order. This results in a branching hierarchy denoted as $H$. The hierarchy plays a key role in the final step to obtain traits concerning each type of roots.

We first identify the path of skeleton edges capturing the stem using the heuristic of [23], as already done in the previous step. This path is called a *stem path*. We then consider all skeleton edges within a cylindrical region around the stem path, where the radius of the cylinder varies along the stem path and is set to be 1.6 times the thickness measure stored on the skeleton vertices on the path. These skeleton edges make up the *stem region*. The factor 1.6 was chosen empirically to ensure that all skeleton junctions representing the beginning of nodal roots are included in the stem region. Both stem path and stem region are labelled as hierarchy level 0.

To identify nodal roots and lateral roots of different orders, we make the following two assumptions, which are shared by DynamicRoots and have been supported by studies of root systems [7, 19, 24]. First, roots higher up in the hierarchy are generally longer. For example, nodal roots are generally longer than 1st-order lateral roots, which in turn are generally longer than 2nd-order lateral roots, and so on. Second, the maximum number of hierarchy levels in a root system is generally kept low. With these assumptions, we developed a heuristic that minimizes the depth of the hierarchy while favoring longer roots higher up in the hierarchy.

**Fig. 6:**
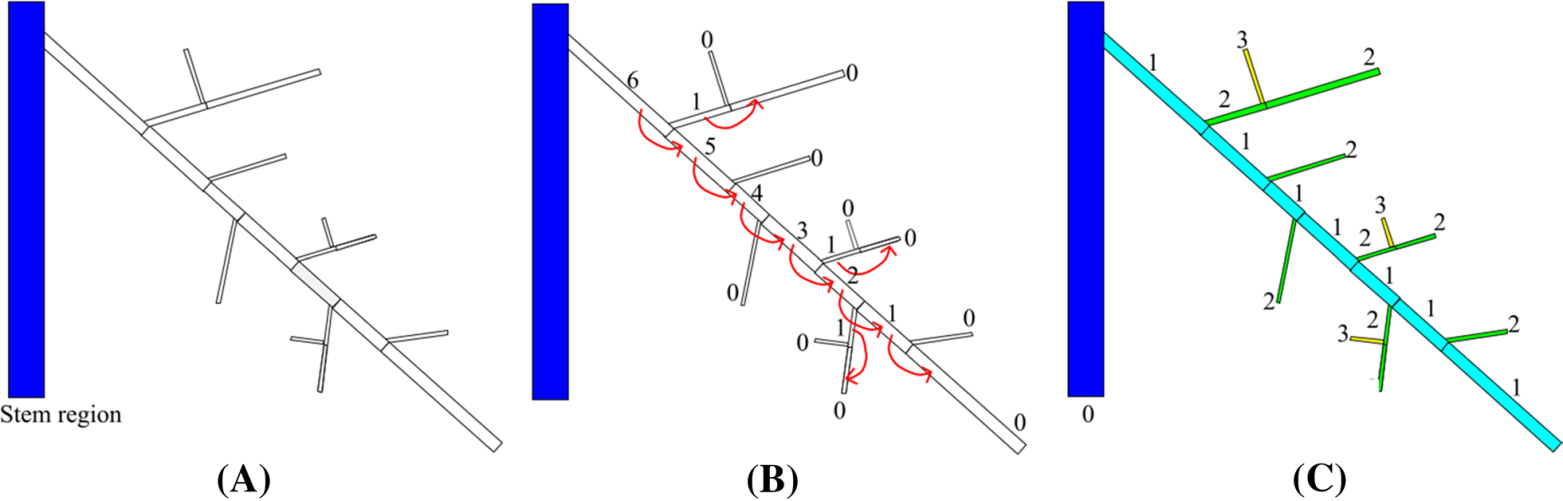
Illustration of the heuristic for inferring hierarchy. **A** The input skeleton $S$ with only the stem region labelled (blue). **B** The first stage associates each parent branch with one of its children branches (indicated by arrows). The numbers are the depth $d(b)$ stored at each branch $b$, an intermediate quantity used to determine the parent–child association. **C** The second stage assigns hierarchy levels (shown as numbers and grouped by colors) to each skeleton branch segment based on the parent–child association. These level labels make up the hierarchy $H$

Our heuristic proceeds in two stages, a bottom-up traversal of the skeleton and then a top-down traversal. They are illustrated in Fig. 6 using a cartoon example. We start with a skeleton $S$ labelled only by the stem region (Fig. 6A). Recall that a branch is a sequence of skeleton edges between two junctions or between a junction and an end vertex. Since $S$ has no cycles, it is a “tree”. We consider the stem region as the “root” of this tree, and this induces a partial ordering on the skeleton such that each junction (outside the stem region) is incident to exactly one *parent branch* and two or more *children b*ranches. The first stage of the heuristic computes, at each skeleton junction, the association between the parent branch with one of the children branches as the continuation of the same root (see arrows in Fig. 6B). The association is computed by visiting the skeleton branches from the leaves of the skeleton tree towards the stem and updating a depth *d(b)* (numbers in Fig. 6B) and a distance *l(b)* at each visited branch $b$, as follows. First, for each branch $b$ incident to an end vertex, we set *d(b)* = 0 and *l(b)* as the length of $b$. We then iteratively visit parent branches whose children branches have already been visited. For a parent branch *b* whose children are *b*_*1*_*, …., b*_*n*_, we associate $b$ with the child ${b}_{i}$ that has the maximal depth $d\left({b}_{i}\right)$. If multiple children have the same maximal depth, $b$ is associated with the ${b}_{i}$ with maximal length $l\left({b}_{i}\right)$. We then set *d(b)* = *d(b*_*i*_*)* + *1* and *l(b)* to be *l(b*_*i*_*)* plus the length of $b$. In the second stage, we visit all branches from the stem to the leaves and assign the hierarchy levels. We assign each branch attached to the stem region a hierarchy level of 1 (i.e., nodal roots). For each parent branch assigned with level $k$, we assign level $k$ to the child branch associated with the parent (computed from the first stage) and level $k+1$ to all other children branches. The resulting hierarchy labelling is shown in Fig. 6C.

### Computing traits

Given the skeleton $S$ and the hierarchy labelling *H*, TopoRoot computes a suite of coarse-grained and fine-grained traits. Like existing works (e.g. [14]), we compute global traits which are aggregated over all roots regardless of their location in the hierarchy, including the total root length, number of roots, and average root length. For fine-grained traits, for each hierarchy level (e.g., nodal roots, 1st-order lateral roots, 2nd-order lateral roots, etc.), we compute the root count, average and total root length, average root tortuosity, average root thickness, average number of children, and the average emergence, midpoint, and tip angle. We also report the length and thickness of the stem. Some of these traits, such as stem length and per-level angle traits, have not been previously reported by existing tools (including DynamicRoots [16]). Details on how each of these traits is computed can be found in Additional file 1: Table S1.

## Results

### Data preparation

TopoRoot is tested on two sets of maize root images, one consisting of 45 X-ray CT scans of excavated root crowns, and another consisting of 495 synthetic images of simulated maize root systems. For validation, we collected ground truth data of nodal root counts for the CT data set and a variety of fine-grained traits for the synthetic data set.

**Fig. 7:**
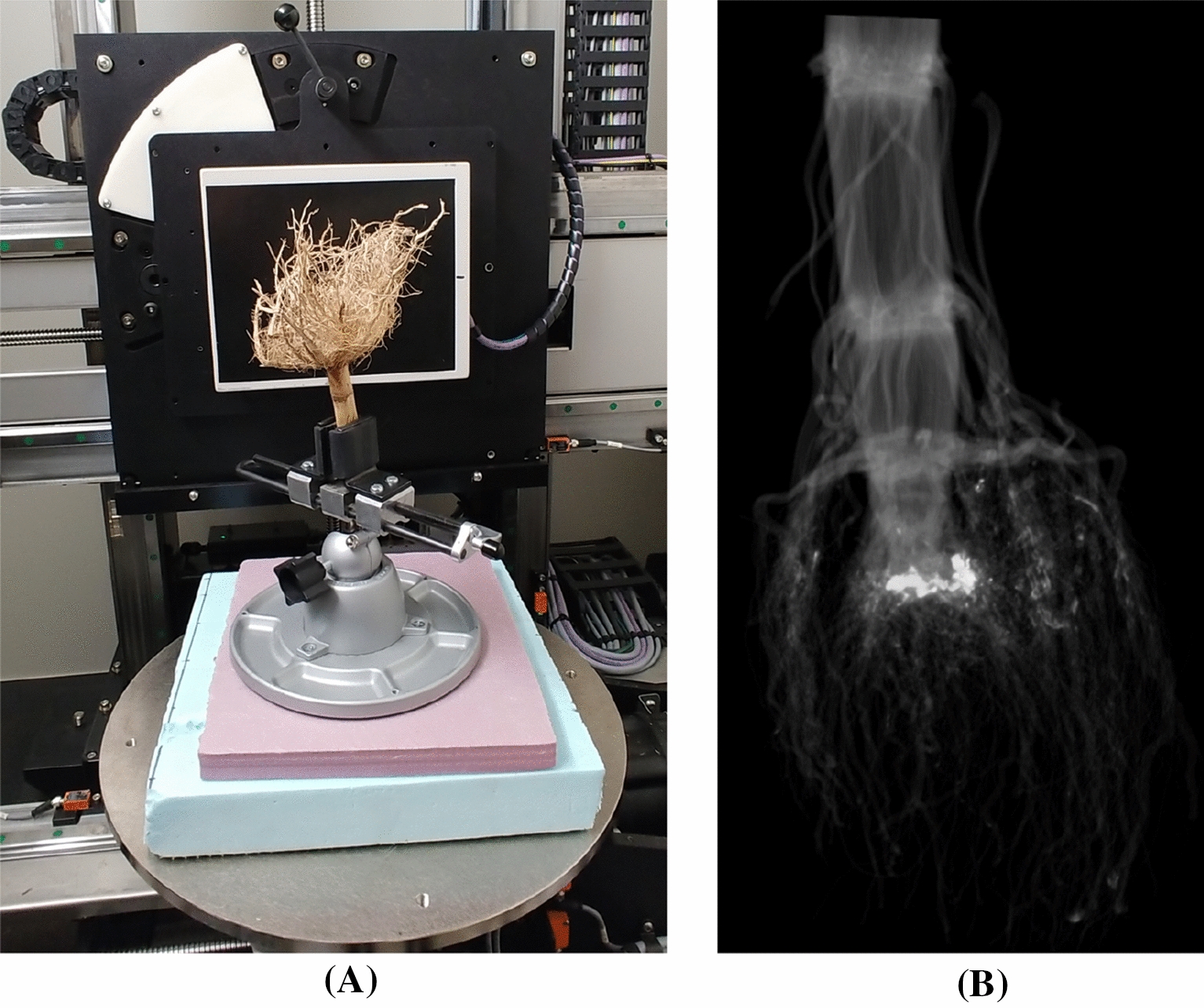
X-ray CT imaging of root crowns. **A** Each maize root crown was clamped and placed on a turntable, which was rotated for 3 min while radiographs were collected at a rate of 10 frames per second. **B** efX-CT software was used to reconstruct a 3D grayscale image from the scan, which is represented as a stack of image slices perpendicular to the z-axis

A cohort of 59 maize seeds were planted in June 2020 at Planthaven Farms in O’Fallon, Missouri (latitude 38.84871204483824, longitude -90.68711352048403) in silt loam soil. The cohort consists of both wild-types (Rt1-2.4 WT) and mutants (Rt1-2.4 MUT) with mutation on the Rootless1 gene, which are known to have decreased nodal root counts [25]. Seeds were planted using jabtype planters into 3.65-m long rows (~ 25.4-cm within row spacing) on 0.9144-m row-to-row spacing. Fields received fertilization with ammonium nitrate. After 54–57 days of growth, the roots were excavated using the Shovelomics protocol [26] in September 2020 and washed to remove large soil chunks (the impact of soil on our method is discussed in the Discussion section). An X5000 X-ray imaging system and efX-DR software (NSI, Rogers, MN) were used to collect X-ray computed tomography (XRT) data (see in Fig. 7). The X-ray source was set to a voltage of 70 kV, current of 1700µA, and focal spot length of 119 μm. Samples were clamped and placed on a turntable for imaging at a magnification of 1.17X and 10 frames per second (fps), collecting 1800 16-bit digital radiographs over a 3 min scan time. efX-CT software was used to reconstruct the scan into a 3D image at 109 μm voxel resolution. This 3D image was exported as a 16-bit RAW file, then ImageJ was used to store the RAW file as a stack of 2D image slices perpendicular to the z-axis. Finally, 14 3D images were removed from the analysis due to excessive soil present in the imaging or missing whorls, resulting in a total of 45 3D images or validating TopoRoot. We performed manual counting of nodal roots for each of the 45 root crowns. Each sample was dissected starting at the highest node (stalk end) moving downward to the root tips. Only attached roots were counted towards the total number of developed roots at each node.

**Fig. 8:**
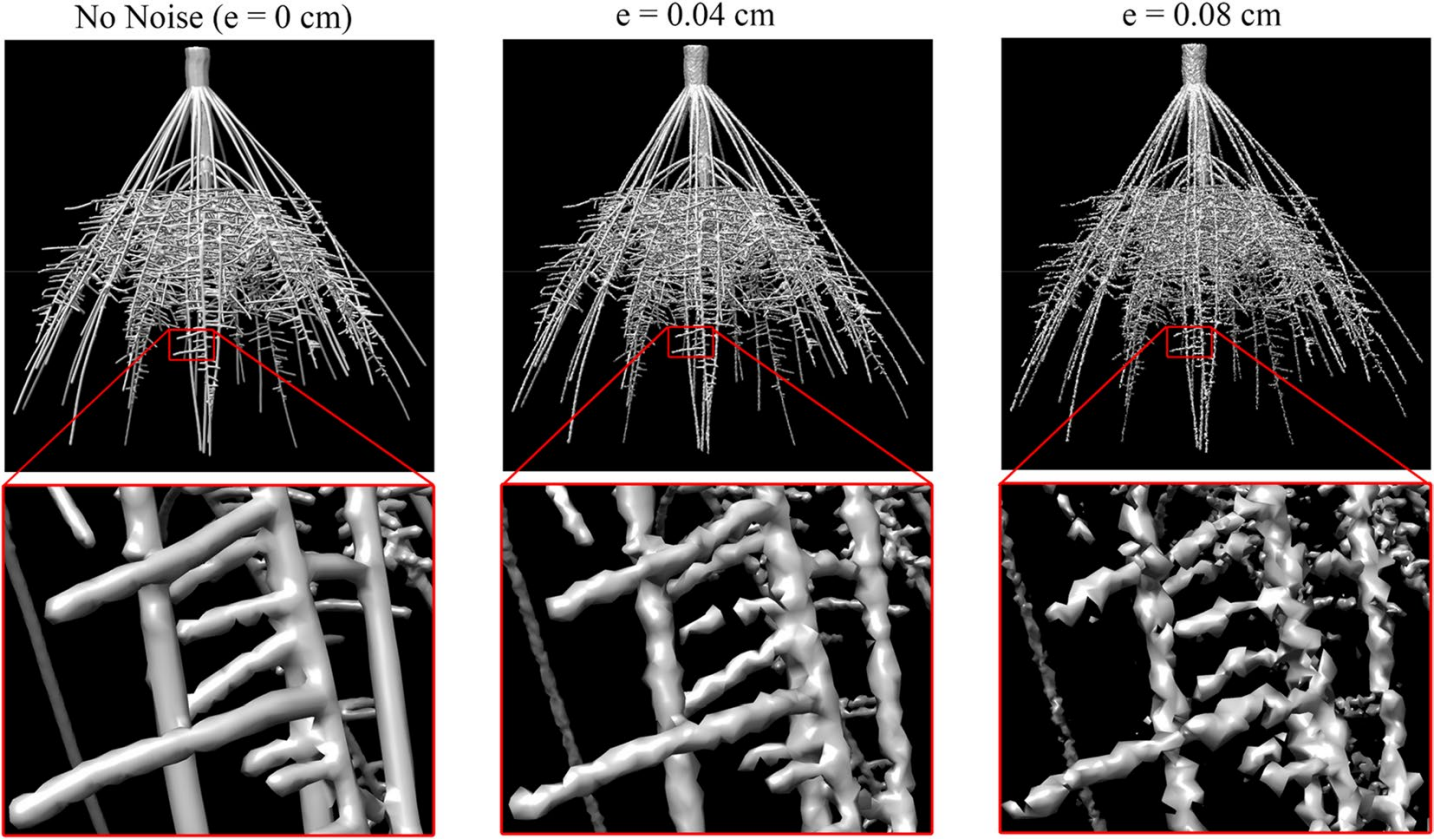
Synthetic maize root images with increasing amounts of noise (e) generated from a simulated root system at 40 days of growth. The closeups show fine lateral roots. With increased noise, the roots exhibit less regular geometry and more topological errors (e.g., disconnections and loops)

It is generally difficult to obtain manual measurement of fine-grained root traits beyond counting the nodal roots. To validate other fine-grained traits produced by our method, we supplement the CT data set with a large benchmark of synthetically generated root images. We adopt OpenSimRoot [19], a highly customizable 3D root growth simulation software that has been widely used in modeling and visualizing root growth [27, 28]. We used OpenSimRoot to create 55 maize root systems ranging in days of growth from 30 to 40 days, numbers of nodal roots ranging from 31 to 69, number of whorls from 5 to 6, and lateral root branching frequency from 0.3 to 0.7 cm / branch. The diameter of the stem was set to be 2 cm, starting diameter for nodal roots is 0.3 cm (gradually decreasing to 0.1 cm after 10 days of growth), lateral roots is 0.04 cm, and fine lateral roots is 0.02 cm. OpenSimRoot provides a detailed hierarchy for each of the simulated roots, from which we obtain the ground-truth traits (roots less than one voxel long in the ground truth model were excluded). For each simulated root system, we synthesize a 512^3 image by computing the signed distance field from the surface of the root using the method of [29] with the inside of the surface having positive values and the outside having negative values. To mimic various levels of image noise, we randomly perturb the distance value at each voxel, with the amount of perturbation ranging from 0 to 0.08 cm in 0.01 increments. This results in 9 images at increasing noise levels for each of the 55 roots, and thus 495 images in total. Figure 8 shows images of one simulated root (at day 40) synthesized at different noise levels. Note that the amount of geometric irregularity and topological noise (e.g., disconnected components and loops) increase with the noise level.

## Experiment settings

The only parameters of TopoRoot that need to be individually tuned for each input image are the three thresholds ($t_{low}$, $t_{mid}$, $t_{high}$) used in the segmentation step. As explained earlier, $t_{mid}$ should be chosen to best capture the overall root shape, $t_{low}$ should be low enough to connect most of the roots, and $t_{high}$ should be high enough to prevent roots from touching. Due to the varying contrast in the CT images, the thresholds tend to vary as well (the values are included in our online data distribution). For the synthetic images, we found that setting $t_{low} = - 0.15,t_{mid} = 0,t_{high} = 0.03$ works well on all images. TopoRoot is implemented in C +  + , and all experiments were run on a Windows 10 machine with an Intel(R) Core (TM) i9-10900X Processor @ 3.70 GHz and 64.0 GB of memory (RAM).

We compared TopoRoot with two previous tools, DynamicRoots [16] (for both global and fine-grained traits) and a 3D version of GiaRoots [30] first published in [14] (for global traits only). We used default parameters for both tools. Since both tools take in a binary segmentation, we ran them after thresholding each image at the threshold $t_{mid}$. To study the impact of topological errors on these tools, we also experimented with first performing the segmentation step of TopoRoot and then running DynamicRoots or GiaRoots on the topologically simplified segmentation (instead of naive thresholding at $t_{mid}$). We call the new protocols DynamicRoots + and GiaRoots + respectively.

### Experimental results: excavated root crowns

**Fig. 9:**
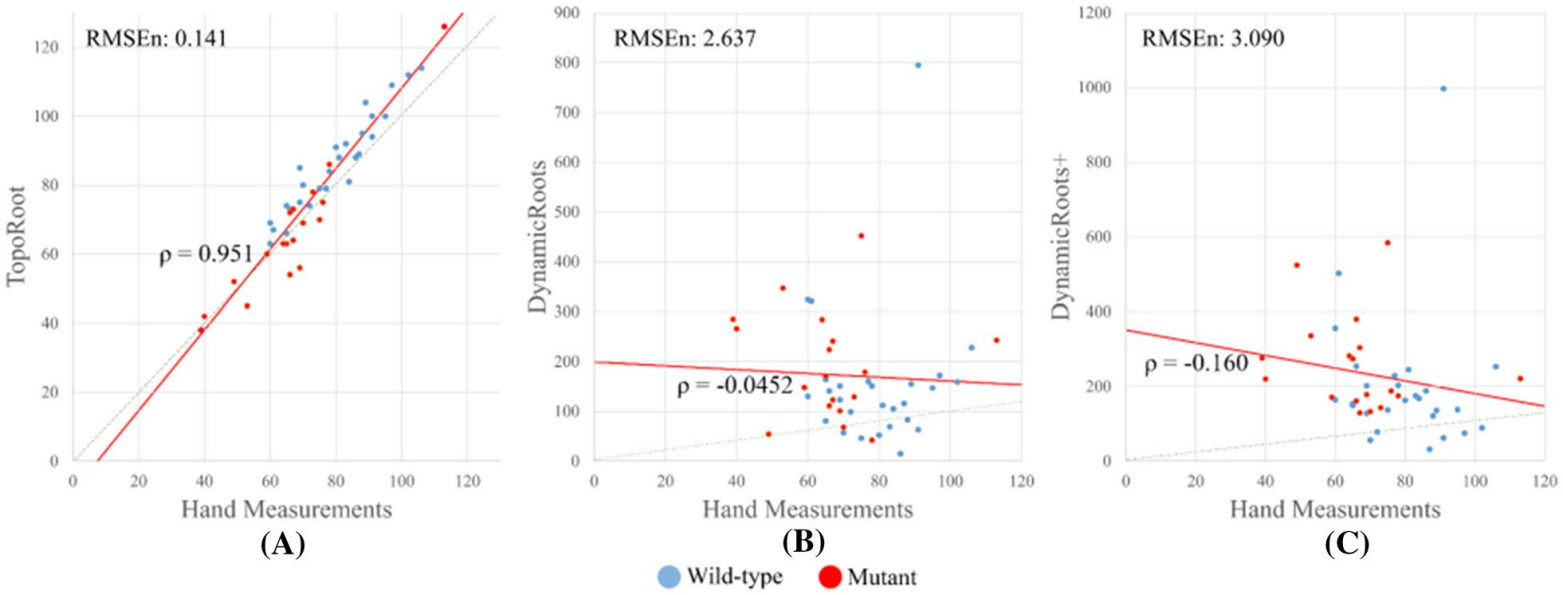
Correlation plots of nodal root count between hand measurements and those obtained by TopoRoot (**A**), DynamicRoots (**B**), and DynamicRoots + (**C**). Blue and red dots indicate wild type and mutant samples. The regression line is in red, and the dashed green line indicates the ideal correspondence between the two measurements. Also reported are Pearson’s correlation coefficients (ρ) and the normalized root mean squared errors (RMSEn)

Figure 9 visually compares the root hierarchies computed by TopoRoot, DynamicRoots, and DynamicRoots + on an example root crown. DynamicRoots produces a point cloud where each point represents an input voxel and is labelled by its hierarchy level (0, 1, 2, etc.). Observe DynamicRoots mis-labelled many roots, such as those highlighted in the black boxes. This is primarily since the methodology of DynamicRoots is designed for much less complex, seedling-stage roots. In addition, a significant portion of the root is missing, as highlighted by the red box. This is because DynamicRoots takes in the naively thresholded image, which has many disconnected parts. Although performing the topological simplification step in TopoRoot allows DynamicRoots + to recover a more complete root shape, mislabelling of hierarchy levels remains (black boxes). In contrast, TopoRoot produces a more visually plausible hierarchy separating the stem (region), nodal roots, and lateral roots.

**Fig. 10:**
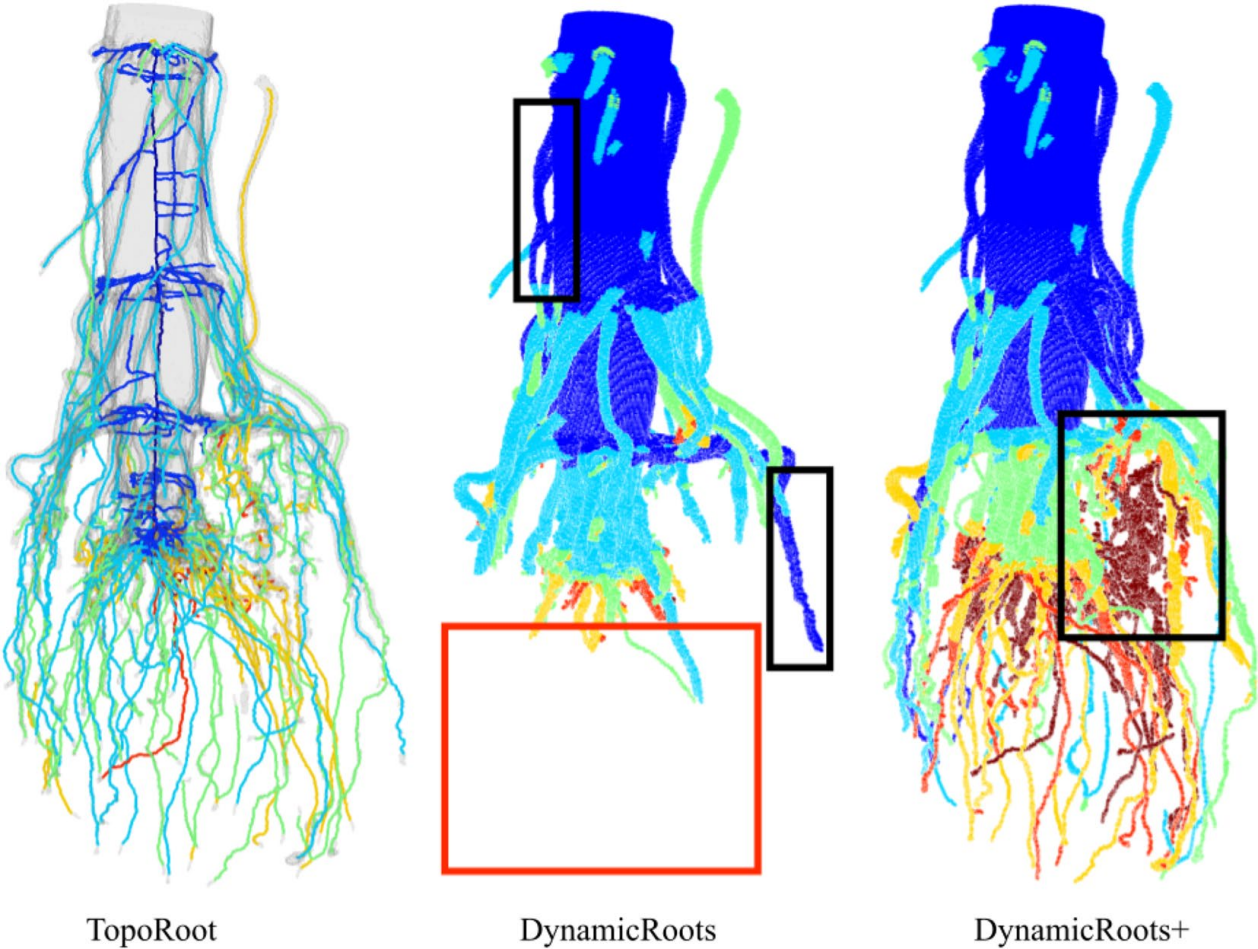
Visual comparison of root hierarchies computed by TopoRoot, DynamicRoots and DynamicRoots + from the X-ray CT scan of an excavated maize root crown. Hierarchy levels are colored as follows: 0 (stem): dark blue, 1 (nodal roots): light blue, 2 (1st-order lateral roots): green, 3 (2nd-order lateral roots): orange, 4 (3rd-order lateral roots): red, ≥ 5: dark red. Black boxes highlight incorrect levels obtained by DynamicRoots and DynamicRoots + , and the red box highlights a missing component in DynamicRoots

We next compare the nodal root count obtained by various tools and by hand measurement. Figure 9 plots the per-sample hand-measured nodal root counts and those computed by TopoRoot (A), DynamicRoots (B) and DynamicRoots + (C) for all 45 samples. The plots also report Pearson’s correlation coefficients ($\rho$) and the normalized root mean squared errors (RMSEn). TopoRoot exhibits a much higher correlation ($\rho =0.951$) and lower error (RMSEn = 0.141) than either DynamicRoots ($\rho =-0.0452$, RMSEn = 2.637) or DynamicRoots + ($\rho =-0.160$, RMSEn = 3.090). We also computed the per-sample relative error of a tool as the ratio of the difference between the tool’s and the hand measurements over the hand measurement, and found that TopoRoot exhibits a much lower mean and standard deviation ($\sigma$) of the relative error (mean = 8.3%, $\sigma =$ 5.6%) than either DynamicRoots (mean = 159.5%, $\sigma =$ 190.0%) or DynamicRoots + (mean = 235.4%, $\sigma =$ 244.4%). The significant over-counting of DynamicRoots + is mostly caused by the mislabeling of nodal roots as level-0 roots, as shown in Fig. 10, which leads to many lateral roots being labelled as level-1 roots. Furthermore, both the nodal root counts computed by TopoRoot and the hand measurements exhibited a significant difference between the mutant and wild type samples, as measured by the independent two-sided Wilcoxon rank sum test (p = 0.00013 for TopoRoot, p = 0.00349 for hand measurements). Neither DynamicRoots (p = 0.126) nor DynamicRoots + (p = 0.0199) showed a significant difference between the mutant and wild-type. This shows that TopoRoot can be useful for differentiating the root system architecture between these two varieties.

### Experimental results: simulated roots

**Fig. 11:**
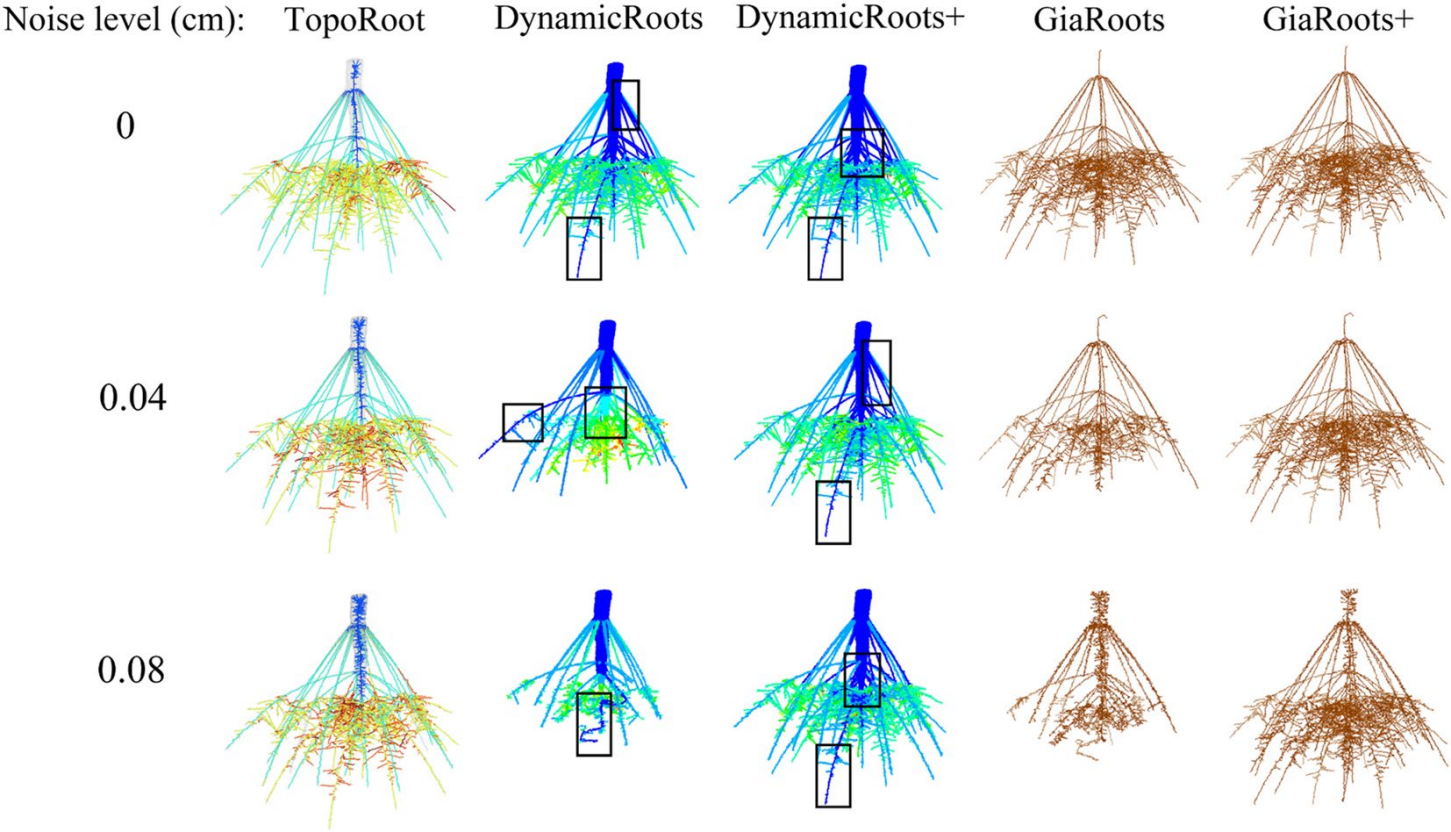
Comparing hierarchies and skeletons computed by different tools from images synthesized at increasing noise levels from a simulated maize root. Hierarchy levels 0, 1, 2, 3 and 4 produced by TopoRoot and DynamicRoots/DynamicRoots + are colored dark blue, light blue, green, orange, and red. The voxelized skeletons produced by GiaRoots/GiaRoots + are colored brown. Black boxes highlight mislabeling of nodal roots as level 0 roots by DynamicRoots and DynamicRoots +

Figure 11 visually compares the root hierarchies produced by TopoRoot and DynamicRoots/DynamicRoots + as well as the voxelized skeletons produced by GiaRoots/GiaRoots + on three synthetic root images at different noise levels (0, 0.04 cm, 0.08 cm). This root system is simulated to be 34 days old, with five whorls, 34 nodal roots, and a lateral root branching frequency between 0.3 and 0.7 cm / branch. As the noise level increases, DynamicRoots and GiaRoots miss more root parts, whereas TopoRoot as well as the extended protocols, DynamicRoots + and GiaRoots + , retain much of the root shape. Observe that, like the CT dataset, the hierarchies produced by DynamicRoots + incorrectly label many nodal roots as level 0 (black boxes). In contrast, the hierarchies produced by TopoRoot are more visually plausible.

We report the mean and deviation of the relative errors of these tools for each fine-grained or coarse-grained trait in Tables 1, 2, 3, 4 (GiaRoots/GiaRoots are considered for global traits only). In Additional file 1: Figures S1–S4, we take a closer look at the accuracy of TopoRoot and the other tools as a function of the noise level of the input images. In general, we observe that higher image noise leads to larger mean errors and/or greater variance by TopoRoot. For most of the traits, TopoRoot maintains a lower error than other tools across all noise levels. We next discuss the accuracy of various traits in detail.

As the base of the hierarchy, the stem traits are among the most accurate (Table 1). As the noise increases, portions of the stem region are lost, resulting in a thinner stem (Additional file 1: Figure S1). Increased noise also causes the stem to wiggle more in the direction perpendicular to its main path, resulting in an increased stem length.

**Table 1:** Accuracy of TopoRoot for stem traits

Trait	≤ *e*	TopoRoot (%)
Stem length	0.04	6.9 (σ = 6.9)
	0.08	7.7 (σ = 7.3)
Stem thickness	0.04	11.9 (σ = 4.3)
	0.08	15.3 (σ = 5.8)

Each entry gives the mean relative error and standard deviation ($\sigma$) for our method across all simulated models and across all noise levels up to *e* = 0.04 cm and e = 0.08 cm

**Fig. 12:**
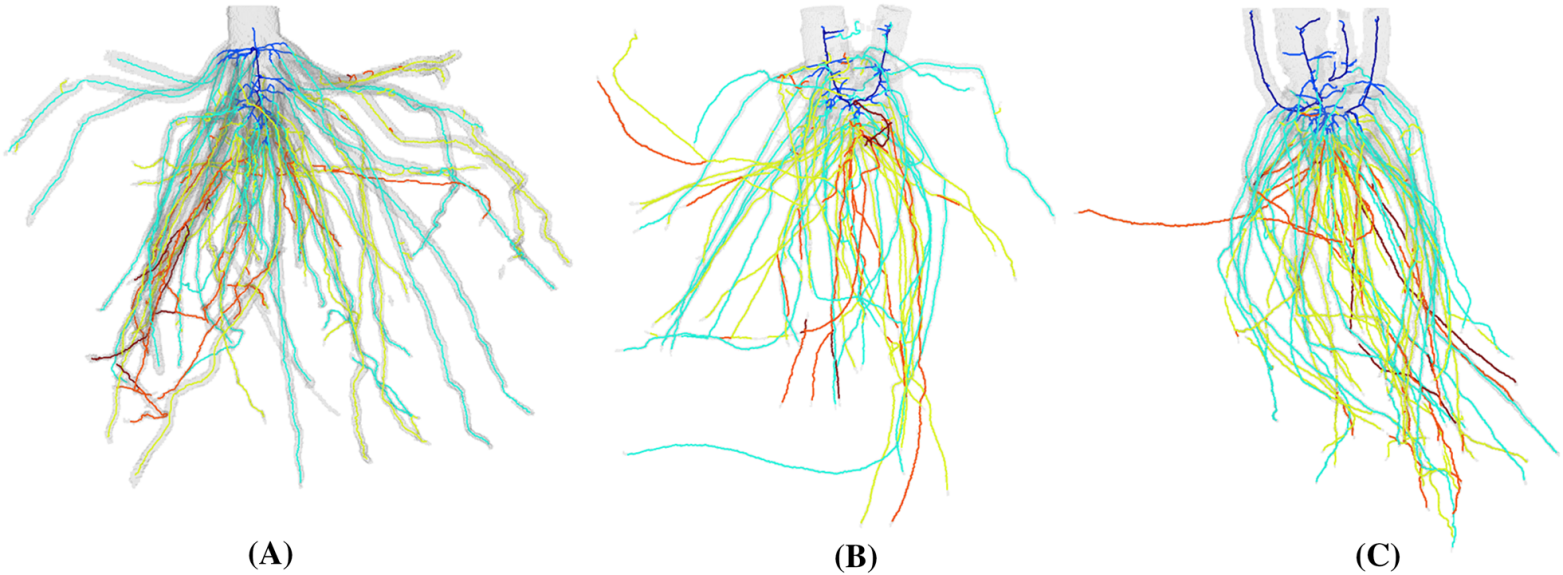
Hierarchies of sorghum roots computed by TopoRoot, showing one tiller (**A**), two tillers (**B**), and four tillers (**C**). Hierarchy levels 0, 1, 2, 3 and 4 are colored dark blue, light blue, green, orange, and red.

Among the nodal root traits (Table 2), the most accurate ones are the root count (mean error 8.3% up to noise level *e* = 0.04, 10.3% up to *e* = 0.08) and emergence/midpoint angles (mean error 5.7/7.1% up to *e* = 0.04, 9.1/9.5% up to *e* = 0.08). The lowest accuracy is seen for the number of children (mean error 40.0% up to *e* = 0.04, 48.6% up to *e* = 0.08) and thickness (mean error 39.2% up to *e* = 0.04, and 38.2% up to *e* = 0.08). These errors are due to misclassifications when the nodal root becomes entangled with higher-order lateral roots. TopoRoot slightly underestimates the average and total length due to faulty cycle breaking and misclassification errors in portions of nodal roots further away from the stem. TopoRoot’s error in nodal root tortuosity is higher than that of DynamicRoots for two reasons. First, the ground truth tortuosity is close to 1 (only for the simulated data, but not for the real maize roots), and DynamicRoots coincidentally produces values close to this because it mistakes many shorter lateral roots as nodal roots, as evidenced by its much shorter average nodal root length and the black boxes of Fig. 12. Second, nodal roots sometimes are misclassified by TopoRoot closer to their tips due to the large number of intersections between roots of different hierarchy levels, resulting in excessive winding. TopoRoot slightly overestimates angle measurements due to misclassification errors further away from the stem which bend the detected paths sideways; these explain the errors in the tip angle measurements.

**Table 2:** Accuracy of TopoRoot, DynamicRoots and DynamicRoots + for nodal root traits

Trait	≤ *e*	TopoRoot (%)	DynamicRoots (%)	DynamicRoots + (%)
Nodal root count	0.04	**8.3** (σ = 8.2)	392.9 (σ = 423.5)	486.2 (σ = 426.6)
	0.08	**10.3** (σ = 10.9)	263.9 (σ = 367.0)	455.2 (σ = 400.0)
Nodal root total length	0.04	**22.4** (σ = 13.1)	46.4 (σ = 17.7)	38.3 (σ = 14.3)
	0.08	**23.9** (σ = 13.7)	56.9 (σ = 19.7)	40.7 (σ = 14.6)
Nodal root average length	0.04	**17.8** (σ = 11.2)	76.6 (σ = 18.8)	80.9 (σ = 16.1)
	0.08	**19.9** (σ = 13.1)	74.0 (σ = 17.4)	81.0 (σ = 15.4)
Nodal root thickness	0.04	39.2 (σ = 27.6)	**36.6** (σ = 33.8)	46.2 (σ = 115.0)
	0.08	**38.2** (σ = 26.8)	50.1 (σ = 150.3)	48.2 (σ = 109.6)
Nodal root number of children	0.04	**36.4** (σ = 21.6)	87.7 (σ = 13.7)	89.4.5 (σ = 12.5)
	0.08	**45.6** (σ = 41.0)	87.0 (σ = 13.1)	89.2 (σ = 12.4)
Nodal root tortuosity	0.04	32.6 (σ = 13.0)	2.3 (σ = 2.5)	**2.0** (σ = 1.8)
	0.08	37.3 (σ = 13.8)	4.5 (σ = 4.4)	**2.9** (σ = 2.3)
Nodal root emergence angle	0.04	**5.7** (σ = 11.9)	N/A	N/A
	0.08	**7.3** (σ = 11.9)	N/A	N/A
Nodal root midpoint angle	0.04	**7.1** (σ = 14.0)	N/A	N/A
	0.08	**9.1** (σ = 13.8)	N/A	N/A
Nodal root tip angle	0.04	**18.3** (σ = 17.7)	N/A	N/A
	0.08	**22.7** (σ = 22.8)	N/A	N/A

Each entry gives the mean relative error and standard deviation ($\sigma$) for a method across all simulated models and across all noise levels up to *e* = 0.04 cm and e = 0.08 cm. For each trait, the method with the lowest mean error is bold-faced

The errors for the lateral root traits (Table 3) are generally larger than nodal root traits, primarily because the imaging noise has a greater impact on the thinner roots more than the thicker ones. There is a greater underestimation of both the total first-order lateral roots and their total length (Additional file 1: Figure S3), due to both the misclassification of the hierarchy levels and the loss of many thin roots in the distance field. On the other hand, the misclassified first-order lateral roots are counted as lateral roots of higher orders, and hence less errors lie in the total lateral root count (mean error 22.2% up to e = 0.04 and 37.0% up to e = 0.08) and lengths (mean error 24.3% up to e = 0.04 and 24.4% up to e = 0.08) over all orders. All methods significantly overestimate the first-order lateral root thickness due to limits in the resolution, but TopoRoot produces the lowest error. The lowest errors are seen in the first-order lateral emergence/midpoint/tip angles (mean error 3.4%/4.1%/5.4% up to e = 0.04 and 3.0%/4.0%/5.9% up to e = 0.08) and tortuosity (mean error 4.6% up to e = 0.04 and 4.5% up to e = 0.08).

**Table 3:** Accuracy of TopoRoot, DynamicRoots and DynamicRoots + for lateral root traits

Trait	≤ *e*	TopoRoot (%)	DynamicRoots (%)	DynamicRoots + (%)
1st-order lateral root count	0.04	**41.4** (σ = 23.4)	75.6 (σ = 11.3)	71.5 (σ = 10.7)
	0.08	**49.0** (σ = 41.5)	80.7 (σ = 11.2)	71.9 (σ = 10.3)
1st-order lateral root total length	0.04	**44.5** (σ = 15.0)	71.9 (σ = 13.9)	66.9 (σ = 15.4)
	0.08	**47.0** (σ = 15.9)	76.7 (σ = 14.9)	67.4 (σ = 14.9)
1st-order lateral root avg. length	0.04	**20.4** (σ = 15.0)	38.1 (σ = 74.6)	26.5 (σ = 54.0)
	0.08	**25.6** (σ = 19.2)	53.2 (σ = 98.2)	26.0 (σ = 52.3)
1st-order lateral root thickness	0.04	**355.0** (σ = 85.8)	424.6 (σ = 101.7)	439.2 (σ = 104.2)
	0.08	**342.8** (σ = 85.3)	427.7 (σ = 112.0)	450.1 (σ = 102.2)
1st-order lateral root number of children	0.04	**68.3** (σ = 34.7)	152.9 (σ = 301.1)	115.6 (σ = 192.7)
	0.08	**80.7** (σ = 50.0)	180.9 (σ = 319.7)	115.6 (σ = 182.6)
1st-order lateral root tortuosity	0.04	**4.6** (σ = 3.2)	11.9 (σ = 2.1)	12.1 (σ = 1.8)
	0.08	**4.5** (σ = 3.2)	10.2 (σ = 3.0)	11.3 (σ = 2.0)
1st-order lateral root emergence angle	0.04	**3.4** (σ = 2.4)	N/A	N/A
	0.08	**3.0** (σ = 2.2)	N/A	N/A
1st-order lateral root midpoint angle	0.04	**4.1** (σ = 2.7)	N/A	N/A
	0.08	**4.0** (σ = 2.7)	N/A	N/A
1st-order lateral root tip angle	0.04	**5.4** (σ = 6.4)	N/A	N/A
	0.08	**5.9** (σ = 7.3)	N/A	N/A
Lateral root count	0.04	**22.2** (σ = 19.5)	60.6 (σ = 13.0)	58.0 (σ = 14.8)
	0.08	**37.0** (σ = 56.1)	66.8 (σ = 12.4)	58.1 (σ = 14.4)
Total lateral root length	0.04	**24.3** (σ = 9.3)	52.3 (σ = 19.6)	50.9 (σ = 19.0)
	0.08	**24.4** (σ = 11.4)	59.1 (σ = 18.8)	56.0 (σ = 17.8)
Average lateral root length	0.04	**20.2** (σ = 14.8)	21.2 (σ = 22.4)	22.0 (σ = 21.6)
	0.08	25.9 (σ = 19.0)	21.4 (σ = 22.9)	**20.6** (σ = 20.2)

Each entry gives the mean relative error and standard deviation ($\sigma )$ for a method across all simulated models and across all noise levels up to *e* = 0.04 cm and e = 0.08 cm. For each trait, the method with the lowest mean error is bold-faced

Finally, combining nodal and lateral roots, TopoRoot produces on average 35.4% relative error (21.5% up to e = 0.04) in the total root count and 25.4% relative error (25.0% up to e = 0.04) in the total root length, which are much lower than DynamicRoots/DynamicRoots + and GiaRoots/GiaRoots + (Table 4). Note that both DynamicRoots and GiaRoots significantly underestimate the root count and total length, even after topological simplification, and the amount of underestimation generally increases with the level of noise (Additional file 1: Figure S4). The only global trait that TopoRoot does not have the lowest error is the average length, due to a combination of DynamicRoots being coincidentally closer due to its underestimation of both the total length and number of roots, and TopoRoot counting an excessive number of roots at higher noise levels. These are the same reasons why the two methods have similar lateral root average length errors.

**Table 4:** Accuracy of TopoRoot, DynamicRoots, DynamicRoots + , GiaRoots, and GiaRoots + for global traits

Trait	≤ *e*	TopoRoot (%)	DynamicRoots (%)	DynamicRoots + (%)	GiARoots (%)	GiARoots + (%)
Total root count	0.04	**21.5** (σ = 18.4)	53.7 (σ = 11.1)	49.3 (σ = 11.3)	57.9 (σ = 13.7)	56.8 (σ = 13.7)
	0.08	**35.4** (σ = 51.8)	60.8 (σ = 12.7)	49.7 (σ = 11.3)	45.7 (σ = 21.1)	57.5 (σ = 13.2
Average root length	0.04	18.4 (σ = 13.5)	9.1 (σ = 11.8)	**9.7** (σ = 9.8)	42.6 (σ = 33.5)	50.2 (σ = 35.3)
	0.08	24.7(σ = 18.8)	**9.9** (σ = 12.5)	13.3 (σ = 12.2)	40.8 (σ = 27.4)	55.6 (σ = 34.2)
Total length	0.04	**25.0** (σ = 8.5)	52.2 (σ = 13.9)	48.7 (σ = 13.1)	46.0 (σ = 12.1)	40.1 (σ = 12.1)
	0.08	**25.4** (σ = 9.7)	59.6 (σ = 14.5)	52.8 (σ = 12.4)	45.7 (σ = 19.6)	38.2 (σ = 13.1)

Each entry gives the mean relative error and standard deviation ($\sigma$) for a method across all simulated models and across all noise levels up to *e* = 0.04 cm and e = 0.08 cm. For each trait, the method with the lowest mean error is bold-faced

## Discussion

A gap exists in the phenotypic measure of root system architecture between fine-grained analyses that can be conducted on entire seedling root systems in laboratory settings, and much coarser global analyses available to field researchers. Since root systems are an emergent property of their many hundreds, thousands, or tens of thousands of constituent roots, this gap is a major hindrance to a comprehensive understanding of root system development, environmental interaction, and the genetics that influence these processes. In previous work, we showed that when global 3D analysis of field excavated maize root crowns was compared to 3D seedling analysis in gellan gum, genetically encoded differences were consistent despite major differences in developmental stage and the growth environment. Whereas DynamicRoots was previously developed for fine scale measurements of 3D seedling root systems containing dozens to hundreds of roots, no similar tool existed for more complex mature root crowns, containing hundreds to thousands of roots. The orders of magnitude of increased complexity motivated unique solutions using both state-of-the-art techniques in computer graphics [20–22] and novel algorithms which eventually led to the development of TopoRoot. While we consider this first version as the foundation of several future planned advancements, discussed below, we were able to present here unprecedented fine-grained analysis of complex, field-excavated 3D root crowns (on average containing 943 total roots [maximum of 2514], 78 nodal roots [maximum of 126], 865 lateral roots [maximum of 2414]) that facilitates “apples to apples” comparisons with existing seedling phenotyping pipelines.

### Error analysis

The steps of our pipeline that are most prone to errors are segmentation and skeletonization. While the algorithm of [20] is very effective in reducing the topological complexity, it may occasionally do so at the cost of altering the structure of the roots (e.g., by breaking a thin root or merging two nearby roots), thereby introducing errors in the hierarchy and fine-grained traits. The problem can be alleviated by pushing thresholds ${T}_{low},{T}_{high}$ closer to ${T}_{mid}$, thus limiting the amount of changes that the algorithm can make. This solution, however, will increase the number of topological errors in the segmentation that need to be resolved in the skeletonization stage. On the other hand, our method for extracting a connected and cycle-free skeleton has several limitations itself. First, we only consider the largest connected component of the skeleton and hence would miss any root parts that are not connected to the main roots in the segmentation stage. Second, our hand-crafted weights ($w(a)$) on the graph arcs in the cycle-removal algorithm may not correctly distinguish between real and false junctions, and as a result the algorithm may detach branches from junctions in the wrong places. Third, the MST formulation of cycle removal cannot recover branches that are already broken in the segmentation stage. These and other limitations all lead to downstream errors in hierarchy and trait analysis. Improving the accuracy of these two steps calls for development of more robust and shape-aware algorithms for topological simplification and tree extraction from a skeleton.

Our method can be sensitive to the amount of soil attached to the roots being imaged. The method works well when the roots are reasonably clean and free of large dirt chunks. Small dirt particles are expected to remain after washing, and our method is designed to handle images containing localized topological noise caused by the dirt particles. If the root contains large chunks of dirt, and due to the similarity in intensity between the dirt and roots, a naive segmentation method (such as thresholding) may create many false and/or cluttered roots. Our method cannot fix these large-scale errors and will produce incorrect hierarchies and traits. Besides more thorough cleaning, an alternative solution is to employ advanced image segmentation methods that are capable of separating soil from roots prior to the application of our method (see more discussions in "Extensions" section).

### Running time

On average, TopoRoot completes in 7 min and 13 s for each sample in the CT scan dataset (downsampled by a factor of 4 to the resolution of 369 × 369 × 465). Since this is much shorter than the time spent imaging and reconstructing one sample, TopoRoot is well suited for high-throughput analysis. The computation time is dominated by the first two steps, topological simplification (3 min and 6 s) and skeletonization (3 min and 44 s). The time complexity of both these steps may increase quickly with the image resolution. For example, running TopoRoot on the original CT images downsampled by a factor of 2, which results in 3D images of resolution 737 × 737 × 931, would take 63 min and 39 s, with 32 min and 25 s spent on topological simplification and 29 min and 9 s on skeletonization. On the other hand, we have not observed a notable improvement in the accuracy of the nodal root count for this data set with the increased image resolution.

### Extensions

In addition to the per-level traits reported in this work, the hierarchy obtained by TopoRoot potentially enables computation of other fine-grained traits. For example, we are currently exploring the use of the hierarchy for computing whorls and the soil plane, which would in turn enable computation of traits such as inter-whorl distances, per-whorl measurements, and the numbers of nodal roots above and below the soil. Preliminary experiments show promising results of whorl detection by clustering the nodal roots branches along the stem path of the skeleton. The soil plane can be potentially identified by the emergence of a large cluster of 1st-order lateral roots along the direction of the stem.

While TopoRoot is designed for high-contrast images (e.g., CT scans) of maize roots, it can be adapted to other types of root systems and images. For root crowns with multiple tillers (e.g., sorghum), we offer a mode of TopoRoot which extends the stem-detection heuristic (during the skeletonization step) by producing a stem path within each region of the skeleton above a given thickness threshold. Preliminary visual experiments show that TopoRoot’s multiple-tiller mode produces plausible hierarchies at a quality like that seen in the single-tiller mode (Fig. 12). Further expanding the stem-detection heuristic to identify the primary root would make the pipeline applicable to taprooted systems as well. Finally, TopoRoot can be extended to work on 3D images that lack a sufficiently high contrast between the roots and the surroundings (e.g., soil), such as in situ imaging of growing roots, provided that the roots can be segmented from its surroundings using a third-party image segmentation algorithm. Examples of such algorithms include region-growing [1], tracking tubular features [31, 32], deep learning [33, 34], and semi-automatic annotation [12, 35]. Some of these methods (e.g., deep learning) produce a probability density field, which can be fed into TopoRoot as the input 3D grayscale image. Others (e.g., region growing) produce a binary 3D image, and TopoRoot can be applied after converting the binary 3D image into a Euclidean distance field (e.g., using [29]).

### Software availability

TopoRoot is available for free at: https://github.com/danzeng8/TopoRoot.

Included in the page are instructions to run the software, and details on the formats of the input and output files. Currently, the accepted inputs are either image slices (suffixed with.png) or.raw files, with a.dat accompanying the.raw file to specify the dimensions. The output consists of a skeleton, a hierarchy annotation of the skeleton, and a spreadsheet of root traits. TopoRoot is currently configured to build and run on Windows 10 machines. A graphical user interface is also available in that repository for visualizing the skeleton and the hierarchy.

## Conclusions

We introduced TopoRoot, a high-throughput method for computing the hierarchies and fine-grained traits from 3D images of maize roots. TopoRoot specifically addresses topological errors, which are common in segmenting 3D images and are barriers for obtaining accurate root hierarchies. Our method combines state-of-the-art methods developed in computer graphics with customized heuristics to compute a wide variety of traits at each level of the root hierarchy. When tested on both 3D scans of excavated maize root crowns and synthetic images of simulated root systems with artificially added noise, TopoRoot exhibits superior accuracy over existing tools (DynamicRoots and GiaRoots) in both coarse-grained and fine-grained traits. Furthermore, the efficiency and automation of TopoRoot makes it suited for a high-throughput analysis pipeline. The results are readily compatible with the Root System Markup Language (RSML; [36]), and major plant structural–functional modelling frameworks such as CRootBox [37] and OpenSimRoot [19].

## Supplementary Information

**Additional file1: Table S1.** TopoRoot’s computed traits. **Fig. S1**. Box plots of relative errors in stem traits computed by TopoRoot. Each box shows the quantiles of relative errors over all 55 synthetic samples at each noise level. **Fig. S2**. Box plots of relative errors in nodal root traits computed by TopoRoot, DynamicRoots and DynamicRoots+. Each box shows the quantiles of relative errors over all 55 synthetic samples at each noise level. **Fig. S3**. Box plots of relative errors in lateral root traits computed by TopoRoot, DynamicRoots and DynamicRoots+. Each box shows the quantiles of relative errors over all 55 synthetic samples at each noise level. **Fig. S4**. Box plots of relative errors in global root traits computed by TopoRoot, DynamicRoots, DynamicRoots+, GiaRoots and GiaRoots+. Each box shows the quantiles of relative errors over all 55 synthetic samples at each noise level.

## Data Availability

The X-ray CT scans of all 45 root crowns, along with the threshold values ($t_{low} ,t_{mid} ,t_{high}$) and hand measurements of nodal roots for each sample, are available in the TopoRoot Github repository: https://github.com/danzeng8/TopoRoot. The synthetic images of simulated roots and associated ground truth trait measurements are available from the corresponding author upon request.

## Abbreviations

RSA: Root system architecture
CT: Computed Tomography
