# Supplementary material for: TopoRoot: a method for computing hierarchy and fine-grained traits of maize roots from 3D imaging
Source: Plant Methods. 2021 Dec 13;17:127. doi: 10.1186/s13007-021-00829-z (PMC8667396; doi:10.1186/s13007-021-00829-z)
Supplement: Supplementary file 1 — Additional file1: Table S1. TopoRoot’s computed traits. Fig. S1. Box plots of relative errors in stem traits computed by TopoRoot. Each box shows the quantiles of relative errors over all 55 synthetic samples at each noise level. Fig. S2. Box plots of relative errors in nodal root traits computed by TopoRoot, DynamicRoots and DynamicRoots+. Each box shows the quantiles of relative errors over all 55 synthetic samples at each noise level. Fig. S3. Box plots of relative errors in lateral root traits computed by TopoRoot, DynamicRoots and DynamicRoots+. Each box shows the quantiles of relative errors over all 55 synthetic samples at each noise level. Fig. S4. Box plots of relative errors in global root traits computed by TopoRoot, DynamicRoots, DynamicRoots+, GiaRoots and GiaRoots+. Each box shows the quantiles of relative errors over all 55 synthetic samples at each noise level. [file 13007_2021_829_MOESM1_ESM.docx]

Supplementary Information

**Supplementary Table 1: TopoRoot’s computed traits**

| **Groups** | **Trait** | **Description** |
| --- | --- | --- |
| Global traits | Total length | Sum of the lengths of stem, nodal roots, and lateral roots. |
|  | Number of roots | Total number of roots across all hierarchy levels. |
|  | Average length | Total length divided by number of roots. |
| Stem traits | Average stem thickness | Average thickness of the vertices along the stem path. |
|  | Stem length | Length of the stem path. |
| Per-level traits | Level *n* root count | The number of level *n* roots. |
|  | Total level *n* root length | Sum of the lengths of level *n* roots. The length is the skeleton distance from the beginning of the root to its tip. |
|  | Average level *n* root length | Total level *n* root length divided by level *n*  root count. |
|  | Level *n* root tortuosity | Length of a root (skeleton distance) divided by the Euclidean distance from the beginning to the tip, averaged across all level *n* roots. |
|  | Level *n* root thickness | Thickness associated with the skeleton vertices in the root, averaged across all level *n* roots. |
|  | Number of level *n* root children | Number of level 2 roots divided by number of level 1 roots. |
|  | Level *n* root tip angle | Angle between the stem direction and the vector from the beginning to the tip of a root, averaged across all level *n* roots. |
|  | Level *n* root emergence angle | Angle between the stem direction and the vector from the beginning to 30 vertices along the skeleton of a root, averaged across all level *n* roots. |
|  | Level *n* root midpoint angle | Angle between the stem direction and the vector from the beginning of a root to the halfway point of the root, averaged across all level *n* roots. |
| Aggregated lateral root traits | Total lateral length | Sum of the lengths of lateral roots whose hierarchy level is greater than or equal to 2. |
|  | Number of lateral roots | Number of lateral roots whose hierarchy level is greater than or equal to 2. |
|  | Average lateral root length | Average length of lateral roots whose hierarchy level is greater than or equal to 2. |

**Supplementary Fig. S1**

**
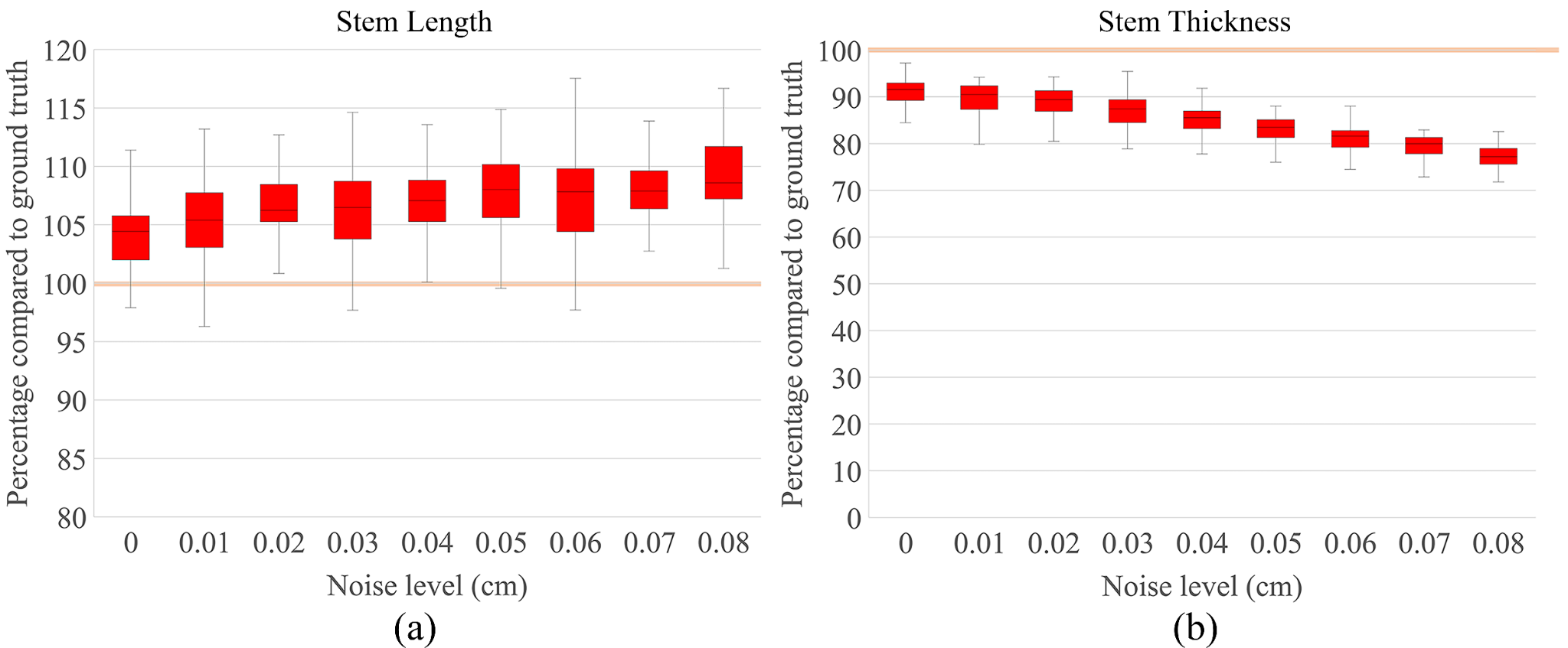
 Box plots of relative errors in** stem traits computed by TopoRoot. **Each box shows the quantiles of relative errors over all 55 synthetic samples at each noise level.**

**Supplementary Fig. S2**

*
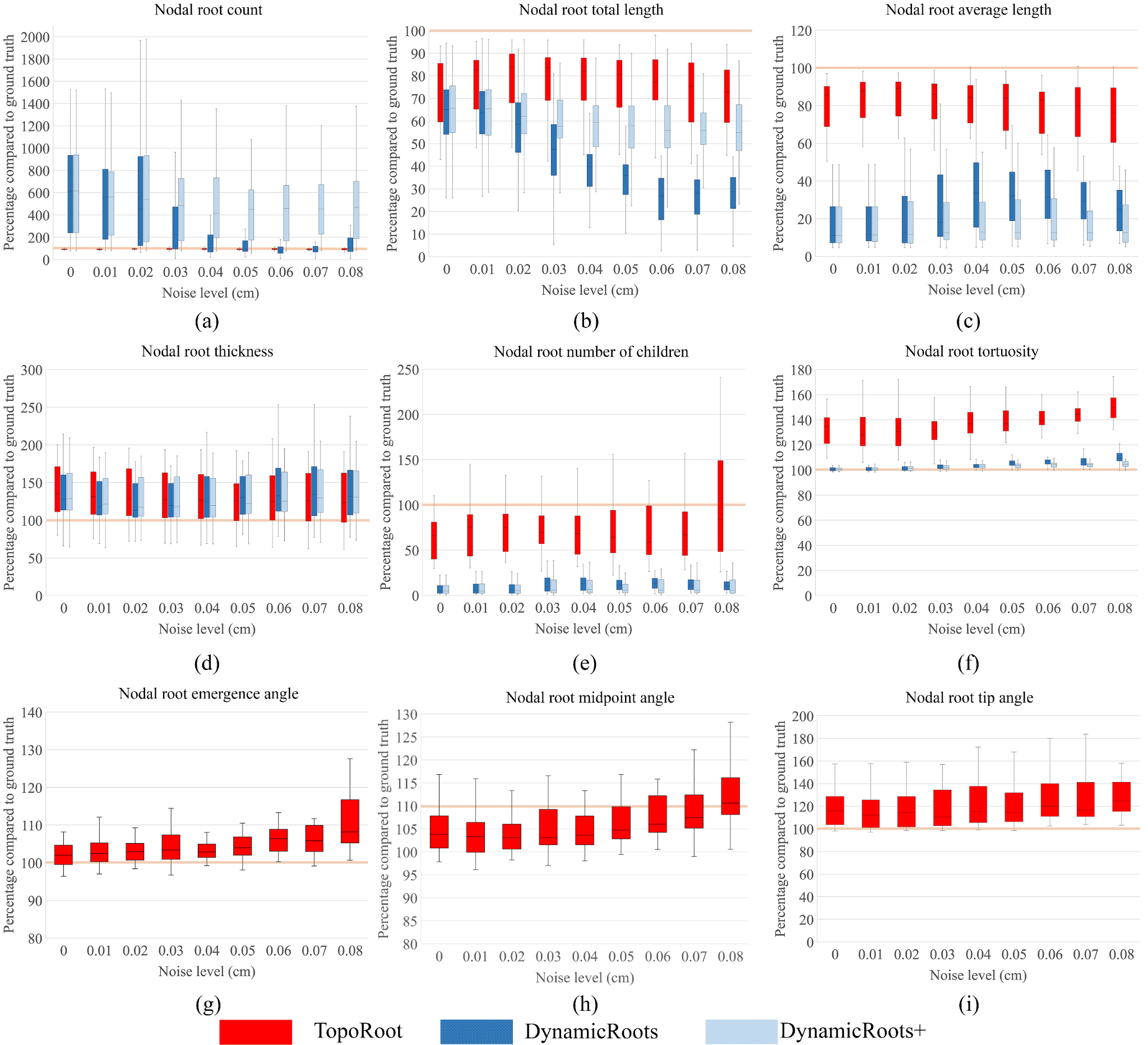
*

Box plots of relative errors in nodal root traits computed by TopoRoot, DynamicRoots and DynamicRoots+. Each box shows the quantiles of relative errors over all 55 synthetic samples at each noise level.

**Supplementary Fig. S3**

*
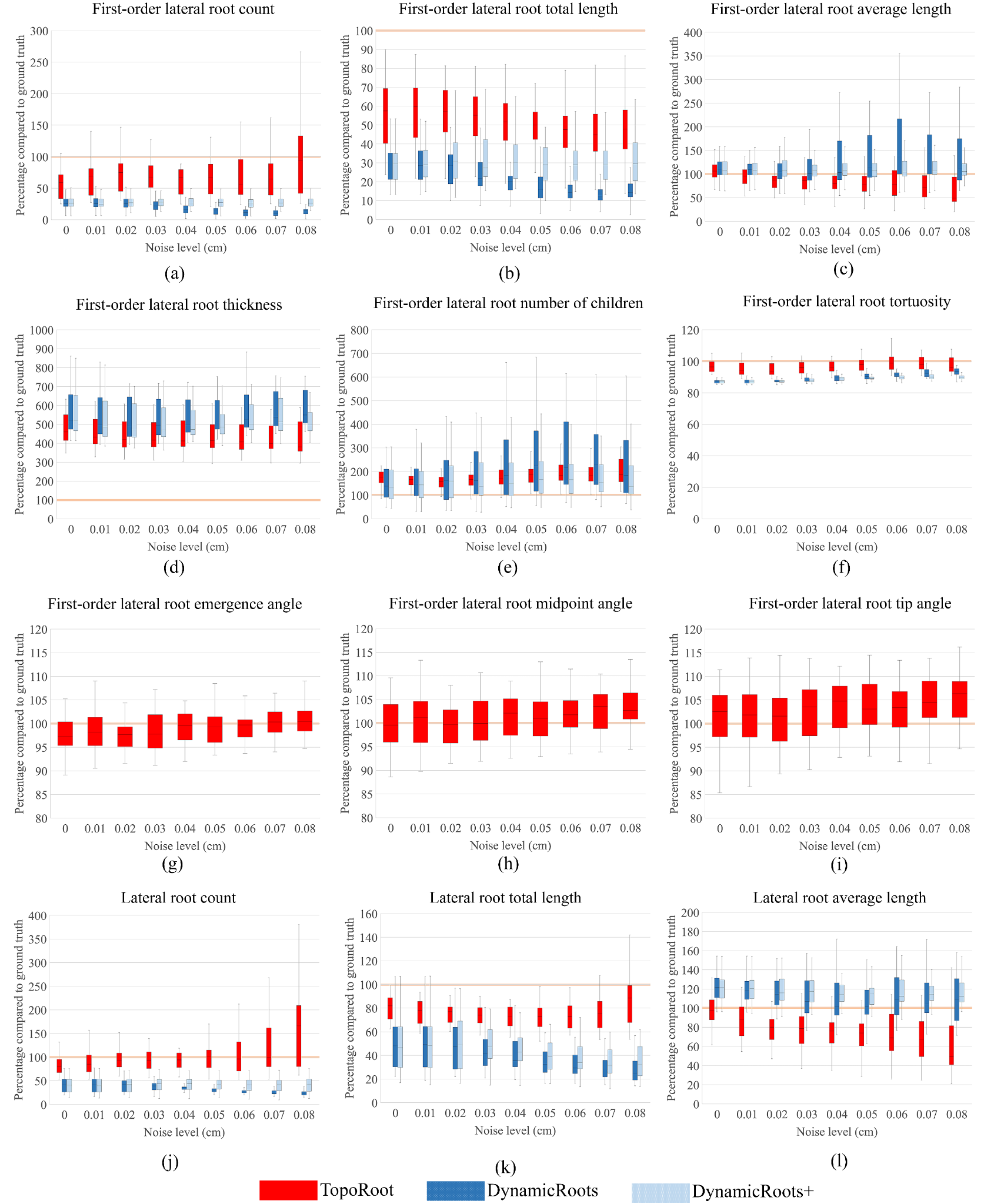
* **Box plots of relative errors in lateral root traits computed by TopoRoot, DynamicRoots and DynamicRoots+. Each box shows the quantiles of relative errors over all 55 synthetic samples at each noise level.**

**Supplementary Fig. S4**


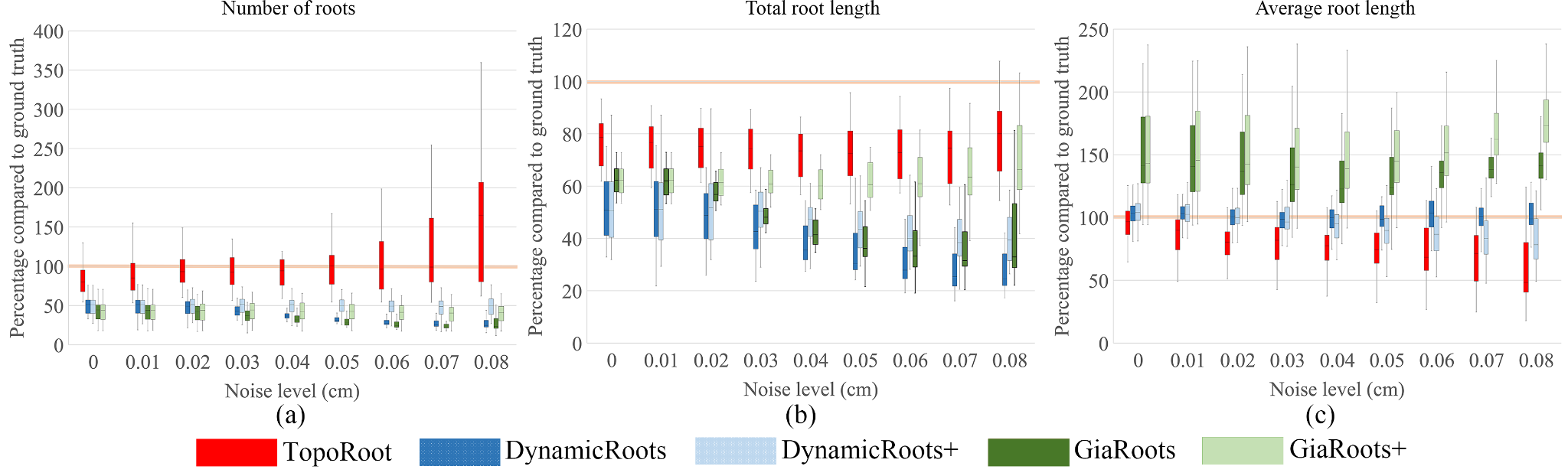


Box plots of relative errors in global root traits computed by TopoRoot, DynamicRoots, DynamicRoots+, GiaRoots and GiaRoots+. Each box shows the quantiles of relative errors over all 55 synthetic samples at each noise level.

To Plant Methods editors: the table below is placed here per the instructions because it exceeds one page. See text for desired location.
